# Dietary Fatty Acids, Macronutrient Substitutions, Food Sources and Incidence of Coronary Heart Disease: Findings From the EPIC‐CVD Case‐Cohort Study Across Nine European Countries

**DOI:** 10.1161/JAHA.120.019814

**Published:** 2021-11-19

**Authors:** Marinka Steur, Laura Johnson, Stephen J. Sharp, Fumiaki Imamura, Ivonne Sluijs, Timothy J. Key, Angela Wood, Rajiv Chowdhury, Marcela Guevara, Marianne U. Jakobsen, Ingegerd Johansson, Albert Koulman, Kim Overvad, Maria‐José Sánchez, Yvonne T. van der Schouw, Antonia Trichopoulou, Elisabete Weiderpass, Maria Wennberg, Ju‐Sheng Zheng, Heiner Boeing, Jolanda M. A. Boer, Marie‐Christine Boutron‐Ruault, Ulrika Ericson, Alicia K. Heath, Inge Huybrechts, Liher Imaz, Rudolf Kaaks, Vittorio Krogh, Tilman Kühn, Cecilie Kyrø, Giovanna Masala, Olle Melander, Conchi Moreno‐Iribas, Salvatore Panico, José R. Quirós, Miguel Rodríguez‐Barranco, Carlotta Sacerdote, Carmen Santiuste, Guri Skeie, Anne Tjønneland, Rosario Tumino, W. M. Monique Verschuren, Raul Zamora‐Ros, Christina C. Dahm, Aurora Perez‐Cornago, Matthias B. Schulze, Tammy Y. N. Tong, Elio Riboli, Nicholas J. Wareham, John Danesh, Adam S. Butterworth, Nita G. Forouhi

**Affiliations:** ^1^ MRC Epidemiology Unit University of Cambridge School of Clinical Medicine Cambridge UK; ^2^ Centre for Exercise, Nutrition and Health Sciences School for Policy Studies University of Bristol Bristol UK; ^3^ Julius Center for Health Sciences and Primary Care University Medical Center Utrecht Utrecht University Utrecht The Netherlands; ^4^ Cancer Epidemiology Unit Nuffield Department of Population Health University of Oxford Oxford UK; ^5^ BHF Cardiovascular Epidemiology Unit Department of Public Health and Primary Care University of Cambridge Cambridge UK; ^6^ Navarra Public Health Institute Pamplona Spain; ^7^ Navarra Institute for Health Research (IdiSNA) Pamplona Spain; ^8^ CIBER of Epidemiology and Public Health (CIBERESP) Madrid Spain; ^9^ National Food Institute Division for Diet Disease Prevention and Toxicology Technical University of Denmark Kongens Lyngby Denmark; ^10^ Department of Odontology Umeå University Umeå Sweden; ^11^ NIHR Cambridge BRC Nutritional Biomarker Laboratory Cambridge UK; ^12^ Department of Public Health Aarhus University Aarhus Denmark; ^13^ Department of Cardiology Aalborg University Hospital Aalborg Denmark; ^14^ Andalusian School of Public Health (EASP) Granada Spain; ^15^ Instituto de Investigación Biosanitaria de Granada (ibs.GRANADA) Granada Spain; ^16^ Universidad de Granada Granada Spain; ^17^ Hellenic Health Foundation Athens Greece; ^18^ International Agency for Research on Cancer World Health Organization Lyon France; ^19^ Section of Sustainable Health Department of Public Health and Clinical Medicine Umeå University Umeå Sweden; ^20^ School of Life Sciences Westlake University Hangzhou China; ^21^ Department of Epidemiology German Institute of Human Nutrition (DIfE), Potsdam‐Rehbrücke Nuthetal Germany; ^22^ National Institute for Public Health and the Environment Bilthoven The Netherlands; ^23^ Le Centre de recherche en Epidémiologie et Santé des Populations (CESP) Faculté de médecine ‐ Univ. Paris‐Sud Faculté de Médecine–UVSQ INSERM Université Paris‐Saclay Villejuif France; ^24^ Gustave Roussy Villejuif France; ^25^ Diabetes and Cardiovascular disease Genetic Epidemiology Department of Clinical Sciences in Malmö Lund University Malmö Sweden; ^26^ Department of Epidemiology and Biostatistics School of Public Health Imperial College London London UK; ^27^ Ministry of Health of the Basque Government Public Health Division of Gipuzkoa Donostia‐San Sebastian Spain; ^28^ Biodonostia Health Research Institute Donostia‐San Sebastian Spain; ^29^ Division of Cancer Epidemiology German Cancer Research Center (DKFZ) Heidelberg Germany; ^30^ Nutritional Epidemiology Unit Fondazione IRCCS Istituto Nazionale dei Tumori Milan Italy; ^31^ Danish Cancer Society Research Center Copenhagen Denmark; ^32^ Cancer Risk Factors and Life‐Style Epidemiology Unit Institute for Cancer Research, Prevention and Clinical Network ‐ ISPRO Florence Italy; ^33^ Department of Clinical Sciences Lund University Malmö Sweden; ^34^ Research Network on Health Services in Chronic Diseases (REDISSEC) Pamplona Spain; ^35^ Dipartmento Di Medicina Clinica E Chiorurgia Federcio II University Naples Italy; ^36^ Public Health Directorate Asturias Spain; ^37^ Unit of Cancer Epidemiology Citta' della Salute e della Scienza Hospital‐University of Turin Turin Italy; ^38^ Center for Cancer Prevention (CPO) Turin Italy; ^39^ Department of Epidemiology Murcia Regional Health Council IMIB‐Arrixaca Murcia Spain; ^40^ Department of Community Medicine University of Tromsø The Arctic University of Norway Tromsø Norway; ^41^ Department of Public Health University of Copenhagen Copenhagen Denmark; ^42^ Cancer Registry and Histopathology Department Azienda Sanitaria Provinciale (ASP) Ragusa Italy; ^43^ Associazione Iblea Ricerca Epidemiologica (A.I.R.E. – ONLUS) Ragusa Italy; ^44^ Unit of Nutrition and Cancer Cancer Epidemiology Research Program Catalan Institute of Oncology (ICO) Bellvitge Biomedical Research Institute (IDIBELL) Barcelona Spain; ^45^ Department of Molecular Epidemiology German Institute of Human Nutrition, Potsdam‐Rehbruecke Nuthetal Germany; ^46^ Institute of Nutritional Sciences University of Potsdam Nuthetal Germany; ^47^ BHF Centre of Research Excellence University of Cambridge Cambridge UK; ^48^ NIHR Blood and Transplant Research Unit in Donor Health and Genomics University of Cambridge Cambridge UK; ^49^ HDR UK Cambridge Wellcome Genome Campus and University of Cambridge Cambridge UK; ^50^ Department of Human Genetics Wellcome Sanger Institute Hinxton UK

**Keywords:** coronary heart disease, dietary guidelines, nutritional epidemiology, primary prevention, saturated fat, Diet and Nutrition, Epidemiology, Primary Prevention, Coronary Artery Disease

## Abstract

**Background:**

There is controversy about associations between total dietary fatty acids, their classes (saturated fatty acids [SFAs], monounsaturated fatty acids, and polyunsaturated fatty acids), and risk of coronary heart disease (CHD). Specifically, the relevance of food sources of SFAs to CHD associations is uncertain.

**Methods and Results:**

We conducted a case‐cohort study involving 10 529 incident CHD cases and a random subcohort of 16 730 adults selected from a cohort of 385 747 participants in 9 countries of the EPIC (European Prospective Investigation into Cancer and Nutrition) study. We estimated multivariable adjusted country‐specific hazard ratios (HRs) and 95% CIs per 5% of energy intake from dietary fatty acids, with and without isocaloric macronutrient substitutions, using Prentice‐weighted Cox regression models and pooled results using random‐effects meta‐analysis. We found no evidence for associations of the consumption of total or fatty acid classes with CHD, regardless of macronutrient substitutions. In analyses considering food sources, CHD incidence was lower per 1% higher energy intake of SFAs from yogurt (HR, 0.93 [95% CI, 0.88–0.99]), cheese (HR, 0.98 [95% CI, 0.96–1.00]), and fish (HR, 0.87 [95% CI, 0.75–1.00]), but higher for SFAs from red meat (HR, 1.07 [95% CI, 1.02–1.12]) and butter (HR, 1.02 [95% CI, 1.00–1.04]).

**Conclusions:**

This observational study found no strong associations of total fatty acids, SFAs, monounsaturated fatty acids, and polyunsaturated fatty acids, with incident CHD. By contrast, we found associations of SFAs with CHD in opposite directions dependent on the food source. These findings should be further confirmed, but support public health recommendations to consider food sources alongside the macronutrients they contain, and suggest the importance of the overall food matrix.

**Nonstandard Abbreviations and Acronyms**
EPIC: European Prospective Investigation into Cancer and Nutrition
GI: glycemic index
MUFA: monounsaturated fatty acid
PREDIMED: Prevención con Dieta Mediterránea
PUFA: polyunsaturated fatty acid
SFA: saturated fatty acid
TEI: total energy intake

**Clinical Perspective**

**What Is New?**

- In a large prospective cohort study including men and women with diverse diets across 9 European countries, there were no strong associations between dietary saturated fatty acids (SFAs) and coronary heart disease (CHD) incidence, or between the substitution of polyunsaturated or monounsaturated fatty acids for saturated fatty acids and CHD incidence.
- In contrast, there were differences in CHD risk when food sources of SFAs were considered, with a lower CHD incidence with consumption of SFAs from fermented dairy products (yogurt and cheese) and fish, but a higher CHD incidence with consumption of SFAs from red meat and butter.

**What Are the Clinical Implications?**

- The differential associations with CHD of SFAs from different food sources provide support for the adoption of a food‐based translation of recommendations for saturated fat intake in dietary guidelines.
- The current findings are based on a large multicountry European study but should be further evaluated in diverse populations where macronutrient intakes, their food sources, and overall dietary patterns vary.

The association of dietary fatty acids with coronary heart disease (CHD) is complex but important because of its enormous public health impact, because diet is a potentially modifiable factor. Most public health dietary guidelines recommend limiting saturated fatty acid (SFA) intake and replacing it with unsaturated fatty acids (specifically polyunsaturated fatty acids [PUFAs]) for the prevention of cardiovascular diseases (CVDs).^1^, ^2^, ^3^ However, meta‐analyses of underpinning randomized trials have drawn different conclusions on the benefits of such dietary modifications for CHD risk.^4^, ^5^, ^6^ Although trials find beneficial effects of substituting PUFAs for SFAs on circulating lipids,^7^ extrapolation of summary trial evidence to public health recommendations is challenging, because this does not take into account foods and nutrients correlated with SFA intakes in free‐living populations. Observational data for longer‐term CHD incidence are inconclusive and dominated by study of populations in the United States and Northern Europe,^8^, ^9^, ^10^ whereas other cohorts that have investigated macronutrient substitutions have generally been unable to confirm their findings.^11^, ^12^, ^13^ Further studies have not modeled the effects of macronutrient substitutions specifically for SFAs^14^ or did not investigate CHD incidence as a separate outcome.^15^, ^16^ Consequently, the role of dietary fatty acids in CHD risk in various populations in Europe, with diverse intake levels and, importantly, food sources, remains uncertain. For example, emerging evidence suggests differing relevance of SFAs from different food sources to CHD,^17^ but studies have yielded mixed results.^11^, ^12^, ^13^, ^18^, ^19^, ^20^, ^21^

Recent appraisals of the health effects of SFAs by the World Health Organization^1^ and the UK Scientific Advisory Committee on Nutrition^2^ have highlighted the need for further research on this topic. For example, considerable gaps in the understanding remain, including the role of monounsaturated fatty acids (MUFAs) as substitution nutrients for SFAs,^9^, ^11^, ^12^, ^22^, ^23^ and lack of clarity on whether potential effects of substituting carbohydrates for SFAs on CHD risk depend on carbohydrate quality.^11^, ^12^, ^15^, ^23^, ^24^, ^25^

To help address these uncertainties, we investigated associations between dietary fatty acids and incident CHD in a large pan‐European prospective case‐cohort study, the EPIC (European Prospective Investigation into Cancer and Nutrition)‐CVD study. Our objectives were to examine (1) associations of dietary total fatty acids, SFAs, MUFAs, and PUFAs with first incident CHD; (2) the relevance of statistically modeled substitution of fatty acids for total carbohydrates, and of MUFAs, PUFAs, and carbohydrates, including by different levels of carbohydrate quality, for SFAs; and (3) associations of SFAs from various food sources with CHD incidence.

## Methods

### Study Population

The EPIC‐CVD study is a case‐cohort study nested within EPIC, a prospective cohort including ~520 000 men and women recruited from 23 study centers in 10 European countries between 1992 and 2000.^26^, ^27^ Requests to access the supporting anonymized data may be made by qualified researchers trained in human participant confidentiality protocols by following the instructions at http://epic.iarc.fr/access/index.php. In this study, we chose a case‐cohort design in EPIC to enable efficient measurement of molecular factors (eg, biomarkers of metabolism, genetics) in a reference subcohort that serves as the comparator group for incidence of several different disease outcomes, including type 2 diabetes mellitus, CHD, stroke, and cancers. The case‐cohort design has the advantages of temporal sequence and power of a cohort study (in that it involves the complete number of incident cases) with the measurement efficiency of a case‐control study.^28^ Among all participants with a stored blood sample (n=385 747), a total of 13 603 incident CHD cases were ascertained during follow‐up, and a random subcohort of 18 249 center‐stratified participants was selected irrespective of future disease status.^26^, ^29^ After excluding participants with prior history of myocardial infarction or stroke, missing follow‐up data, non–first CHD events (where first event was not myocardial infarction), preexisting angina, missing covariates, and participants from Norway because of small sample size, a total of 16 730 random subcohort members and 10 529 participants with an incident CHD event (among whom 572 participants were in the subcohort, by design of a case‐cohort study) were included in the analyses (Figure S1). The study complies with the Declaration of Helsinki. Ethical review boards of the cohorts approved the study protocol, and all participants provided written informed consent.^27^

### Ascertainment of CHD

The primary outcome was first nonfatal or fatal CHD (*International Classification of Diseases, Ninth Edition* [*ICD‐9*] codes 410–414 and *Tenth Edition* [*ICD‐10*] codes I20–I25).^26^ Nonfatal and fatal first CHD events were included as separate secondary outcomes in sensitivity analyses. Methods to ascertain incident CHD events in different centers included self‐report, linkage with registries, review of medical records, or a combination of these. Suspected CHD was validated among all ascertained cases within centers, except in the Netherlands, United Kingdom, and Sweden, where validation was conducted among a sample of CHD events, and France, where no validation information was available. The last year of follow‐up varied between 2003 and 2010 across centers. Nonfatal and fatal events occurring within 28 days of each other were considered a single fatal event. Follow‐up data for each participant were censored at the time of a first CHD event or the end of the follow‐up period, whichever occurred first.

### Assessment of Dietary Intake

Habitual food consumption over the past year was assessed by center‐specific food frequency questionnaires or diet histories, either self‐administered or assessed in face‐to‐face interviews.^27^ Validity of each dietary questionnaire was assessed in subgroups of participants within centers using a reference method of monthly 24‐hour recalls or weighed food records.^30^ In analyses comparing intakes based on the 2 instruments, correlation coefficients were between 0.31 and 0.87 for total fat (except they were lower in Greece, 0.09 in men and 0.26 in women), 0.39 to 0.84 for SFA, 0.28 to 0.89 for MUFA, and 0.26 to 0.89 for PUFA.^30^ All dietary nutrient data were collected in a cohort‐specific manner and standardized centrally within the EPIC consortium. The European Nutrient Database was established to facilitate comparability of the dietary exposures. The European Nutrient Database and estimation of food and nutrient intakes have been described in detail previously.^31^ Mean dietary glycemic index (GI) was calculated using standardized methods.^32^ Dietary total fatty acids were calculated as the sum of SFAs, MUFAs, and PUFAs. Total energy intake (TEI) was defined as daily energy from dietary fatty acids, carbohydrates, and protein from plant, animal, and mixed/unknown origin (in kilocalories). Alcohol was not considered a feasible substitution nutrient, and therefore was not included in the definition of TEI but was used as a model covariate.^9^, ^14^ Macronutrients were expressed as their relative contribution to TEI (%TEI). Dietary fiber and GI were energy adjusted with the residual method for analyses of associations with CHD. Dietary SFAs from the following food groups were expressed in %TEI: total dairy (excluding butter) and subtypes milk, yogurt/thick milk, and cheese; added fats and subtypes vegetable (plant) oils, butter, and margarine; total meat and subtypes red, processed, and poultry; cakes and biscuits; sugar and confectionary; cereals; eggs; condiments and sauces; fish; and nuts and seeds.

### Assessment of Covariates

Lifestyle health behaviors, social factors, and medical history were assessed by country‐specific questionnaires.^27^ Height and weight were measured in most centers except EPIC‐Oxford and France, where self‐reported height and weight were used for most participants for whom measured values were not available.^27^ Methods for measuring other vascular risk factors are detailed in the Table S1 legend.

### Statistical Analysis

All analyses were performed using Stata version 14 (StataCorp College Station, TX). The percentage of individuals excluded because of missing values of baseline covariates among the study sample was <5%, and based on this we had prespecified to conduct a complete‐case analysis. Baseline characteristics were summarized in the subcohort. We estimated Pearson partial correlation coefficients (95% CIs) of dietary SFAs, MUFAs, and PUFAs with (1) all macronutrients, adjusted for age and sex and (2) foods, adjusted for age, sex, TEI, and body mass index, within the subcohort of each country, to allow for country‐specific differences in confounding structures. We then applied the Fisher *z* transformation to these estimates and combined them across countries using random‐effects meta‐analysis. Skewed variables (PUFA, TEI, fiber, triglycerides, total cholesterol: high‐density lipoprotein cholesterol [HDL‐C] ratio, and C‐reactive protein) were log‐transformed where required. Mean (95% CI) vascular risk factors were calculated by subcohort quintiles of dietary fatty acids, adjusted for age, sex, and EPIC center using linear regression to estimate least squares means. In these analyses, no correction for multiple testing was prespecified, but we considered the direction, magnitude and precision (with 95% CIs), as well as consistency, of associations in the interpretation of our findings.

### Modeling Associations with CHD

To account for the oversampling of incident CHD cases in a case‐cohort study design, modified Cox proportional hazards models using Prentice weights were fitted to estimate hazard ratios (HRs) and 95% CIs of total fatty acids, SFAs, MUFAs, and PUFAs with CHD within countries, using age as the underlying time variable and stratifying baseline hazards by sex. To evaluate the effect of confounding on associations, we constructed 4 models with sequential adjustment for different types of potential confounding factors as follows: Model 1 (basic confounders) was adjusted for age at study entry, study center, and TEI (continuous). Model 2 additionally included lifestyle and socioeconomic characteristics: education (low, medium, high), smoking status (never, former, current), and physical activity (inactive, moderately inactive, moderately active, active). Model 3 additionally included dietary factors: dietary fiber (continuous), fruit and vegetable intake (continuous), and alcohol (0, 0–6, 6–12, 12–24, >24 g/d). Model 4 additionally included known cardiometabolic risk factors: body mass index (continuous), and preexisting diabetes mellitus, hypertension, and hyperlipidemia (self‐reported, yes; no/unknown for each). To estimate combined HRs (95% CIs), country‐specific results were pooled using random effects under the assumption that the associations varied by country because of differences in demographic and lifestyle characteristics, and assessment methods. We conducted univariate meta‐analysis and also multivariate meta‐analysis, which accounted for correlations between estimated HRs for all macronutrients and TEI (but not other covariates). Heterogeneity was assessed with the *I* ^2^ statistic. Associations were modeled across quintiles of exposures. After confirming no suggestion of nonlinearity (Table S2), we used the nutrient density model to evaluate the isocaloric substitution per 5%TEI from fatty acids (i.e., as continuous covariates) for any other energy source.^33^

In macronutrient‐specific isocaloric substitution models, we modeled (1) substitution of 5%TEI from total fatty acids, and from SFAs, MUFAs, and PUFAs for 5%TEI from carbohydrates, and (2) substitution of 5%TEI from MUFAs, PUFAs, and carbohydrates for 5%TEI from SFAs, by including TEI and all macronutrients (i.e., total fatty acids or SFAs, MUFAs, and PUFAs; carbohydrates; protein from plant, animal, and unknown/mixed origin) in the models except the nutrient to be substituted for (i.e., carbohydrates or SFAs). In Models 3 and 4 (adjusting for fruit and vegetable intake), associations of carbohydrates should be interpreted as carbohydrates from food sources other than fruit and vegetables.

### Testing for Effect Modification

To evaluate potential effect modification by carbohydrate quality, associations of substituting carbohydrates for SFAs were stratified by thirds of GI^32^ (low: ≤54.4, medium: 54.4–57.5, and high: ≥57.5). Associations were also stratified to evaluate potential effect modification by biological differences (i.e., sex and age [by median, <52.4 versus ≥52.4 years]), plausibility of reported energy intake (suspected under‐, plausible‐, and over‐reporting, defined by intake levels±2 SD of predicted total energy expenditure), and reverse causality (preexisting diabetes mellitus, hypertension, and/or hyperlipidemia). Statistical significance of interactions was evaluated with the *P* value of a cross‐product term of effect modifiers and exposures. Countries with <10 CHD cases or noncases in exposure categories were excluded.

### Food‐Specific SFAs and CHD

First, we analyzed associations with CHD of SFAs (per 1%TEI) from primary food sources previously described in EPIC,^34^ plus yogurt and red meat,^35^, ^36^ and poultry (main meat subtype consumed in EPIC). Second, associations of each food source were additionally adjusted for the sum of SFAs from all other foods. We estimated HRs of substituting macronutrient intakes (MUFAs, PUFAs, carbohydrates, and SFAs from other foods) for SFAs from selected SFA‐rich foods. The selected foods were those where the SFA content showed evidence of association with CHD at *P*<0.05. Given their shared direction of association in our analyses, and the relatively low contribution of yogurt to SFA consumption (Table S3), SFAs from yogurt and cheese were combined into SFAs from fermented dairy products in macronutrient‐specific substitution analyses to increase statistical power.

### Ancillary Analyses

Heterogeneity among geographical regions (South: Greece, Italy, Spain, France; Central: the Netherlands, United Kingdom, Germany; North: Denmark, Sweden) was assessed with the *I* ^2^ statistic and tested against a *χ* ^2^ distribution. We also (1) investigated associations with fatal and nonfatal CHD separately, (2) excluded the first 2 years of follow‐up to evaluate potential reverse causality, (3) excluded participants with extreme energy intakes (<500 or >3500 kcal/d for women; <800 or >4000 kcal/d for men), and (4) Winsorized top and bottom 1% of covariates to evaluate effects of extreme reporting. Because there are alternative approaches to energy adjustment than the nutrient density model we used, we repeated analyses of associations among dietary SFAs, MUFAs, and PUFAs with CHD using 2 further modeling approaches: (1) the nutrient residual model and (2) the energy partition model.^33^ In the nutrient residual model, we evaluated associations per 10 energy‐adjusted grams per day from a specific fatty acid to substitute for any other sources of energy; in the energy‐partition model, we estimated the effect of adding 10 g/d (90 kcal/d) from a specific fatty acid to existing macronutrient intakes by adjusting for all other macronutrients but not for TEI. We conducted post hoc analyses, repeating primary analysis Model 4 pooling data from all countries together in 1‐stage fixed‐effects models, rather than country‐specific 2‐stage random‐effects modeling, to evaluate similarity in results using these 2 different approaches. We also conducted calibration analyses by correcting observed HRs (95% CIs) from food‐frequency questionnaire data for country‐specific regression dilution ratios that were estimated among ~8% of participants with single 24‐hour recall data available.

## Results

### Baseline Characteristics

The median subcohort age was 52.4 years (interquartile range, 46.0–59.2 years), and 62% were women (Table 1). Diets varied by country; for instance, intake levels and ranges of dietary MUFAs were higher in Southern countries (Greece, Spain, and Italy) compared with the rest of Europe (Figure S2). Median (interquartile range) intakes were 35.0 (30.9–39.2) %TEI for total fatty acids, 14.0 (11.7–16.4) for SFAs, 14.1 (11.9–17.0) for MUFAs, and 5.5 (4.5–7.0) for PUFAs (Table 2). SFAs correlated positively with MUFAs and animal proteins, and inversely with carbohydrates and plant proteins (Table S4). Most vascular risk factors varied modestly across quintiles of fatty acids. Comparing top versus bottom SFA quintiles, triglycerides were 0.08 mmol/L higher, whereas non–HDL‐C and total cholesterol:HDL‐C ratios were similar (Table S1).

**Table 1:** Baseline Participant Characteristics in the Lowest (Q1) and Highest (Q5) Quintiles of Dietary Fatty Acid Intake (in Percent of Energy Intake) in the EPIC‐CVD Case‐Cohort Study Subcohort (n=16 730)*

	All	Total Fatty Acids		SFAs		MUFAs		PUFAs	
		Q1, n=3346	Q5, n=3346	Q1, n=3346	Q5, n=3346	Q1, n=3346	Q5, n=3346	Q1, n=3346	Q5, n=3346
Age, y	52.4 (46.0–59.2)	53.3 (47.5–59.8)	50.9 (43.6–58.5)	52.0 (45.5–58.5)	52.9 (47.2–59.8)	54.6 (49.6–60.3)	49.5 (42.9–56.8)	51.9 (45.1–58.9)	52.1 (45.6–59.4)
Women	10 414 (62.2)	2261 (67.6)	2016 (60.3)	2016 (60.3)	2070 (61.9)	2387 (71.3)	2029 (60.6)	2158 (64.5)	2089 (62.4)
Education									
None/primary	7154 (42.8)	1487 (44.4)	1497 (44.7)	1981 (59.2)	1062 (31.7)	1149 (34.3)	1914 (57.2)	1670 (49.9)	1408 (42.1)
Secondary	2531 (15.1)	500 (14.9)	506 (15.1)	404 (12.1)	562 (16.8)	570 (17.0)	501 (15.0)	539 (16.1)	459 (13.7)
Vocational/university	7045 (42.1)	1359 (40.6)	1343 (40.1)	961 (28.7)	1722 (51.5)	1627 (48.6)	931 (27.8)	1137 (34.0)	1479 (44.2)
Physical activity									
Inactive	4084 (24.4)	797 (23.8)	1002 (29.9)	1071 (32.0)	709 (21.2)	605 (18.1)	1144 (34.2)	937 (28.0)	834 (24.9)
Moderately inactive	5614 (33.6)	1095 (32.7)	1093 (32.7)	1083 (32.4)	1154 (34.5)	1030 (30.8)	1123 (33.6)	1167 (34.9)	1082 (32.3)
Moderately active	3806 (22.7)	700 (20.9)	764 (22.8)	632 (18.9)	838 (25.0)	827 (24.7)	674 (20.1)	689 (20.6)	824 (24.6)
Active	3226 (19.3)	754 (22.5)	487 (14.6)	560 (16.7)	645 (19.3)	884 (26.4)	405 (12.1)	553 (16.5)	606 (18.1)
Smoking status									
Never	7912 (47.3)	1723 (51.5)	1449 (43.3)	1731 (51.7)	1374 (41.1)	1713 (51.2)	1587 (47.4)	1690 (50.5)	1550 (46.3)
Former	4425 (26.4)	934 (27.9)	834 (24.9)	845 (25.3)	913 (27.3)	982 (29.3)	730 (21.8)	838 (25.0)	911 (27.2)
Current	4393 (26.3)	689 (20.6)	1063 (31.8)	770 (23.0)	1059 (31.6)	651 (19.5)	1029 (30.8)	818 (24.4)	885 (26.4)
Current drinker	13 856 (82.8)	2619 (78.3)	2764 (82.6)	2429 (72.6)	2923 (87.4)	2748 (82.1)	2572 (76.9)	2612 (78.1)	2779 (83.1)
Alcohol intake, current drinkers, g/d	8.6 (2.5–21.3)	6.4 (1.8–17.2)	9.1 (2.8–22.3)	9.8 (2.1–25.5)	8.5 (2.8–19.5)	5.6 (1.7–13.9)	10.0 (2.4–25.3)	6.9 (1.7–20.8)	9.5 (3.0–22.0)
Menopausal status, women									
Premenopausal	3471 (20.7)	630 (18.8)	807 (24.1)	719 (35.7)	621 (30.0)	538 (22.5)	942 (46.4)	764 (35.4)	738 (35.3)
Perimenopausal	1687 (10.1)	363 (10.8)	309 (9.2)	256 (12.7)	414 (20.0)	401 (16.8)	226 (11.1)	318 (14.7)	347 (16.6)
Postmenopausal	5256 (31.4)	1268 (37.9)	900 (26.9)	1041 (51.6)	1035 (50.0)	1448 (60.7)	861 (42.4)	1076 (49.9)	1004 (48.1)
BMI, kg/m^2^	25.7 (23.2–28.7)	25.7 (23.2–28.5)	26.1 (23.2–29.2)	26.8 (24.2–29.7)	24.8 (22.4–27.6)	25.2 (22.8–27.9)	26.9 (24.2–30.0)	25.6 (23.0–28.6)	25.9 (23.3–29.0)
Overweight, 25–30 kg/m^2^	6654 (39.8)	1380 (41.2)	1301 (38.9)	1491 (44.6)	1164 (34.8)	1292 (38.6)	1432 (42.8)	1306 (39.0)	1307 (39.1)
Obesity, >30 kg/m^2^	2919 (17.4)	550 (16.4)	678 (20.3)	768 (23.0)	424 (12.7)	458 (13.7)	830 (24.8)	582 (17.4)	655 (19.6)
Preexisting diabetes mellitus	514 (3.1)	134 (4.0)	119 (3.6)	158 (4.7)	74 (2.2)	118 (3.5)	119 (3.6)	88 (2.6)	139 (4.2)
Preexisting hypertension	3295 (19.7)	674 (20.1)	656 (19.6)	712 (21.3)	576 (17.2)	651 (19.5)	669 (20.0)	681 (20.4)	706 (21.1)
Preexisting hyperlipidemia	2465 (14.7)	604 (18.1)	464 (13.9)	826 (24.7)	284 (8.5)	454 (13.6)	640 (19.1)	555 (16.6)	550 (16.4)
Country									
Greece	1201 (7.2)	14 (0.4)	931 (27.8)	242 (7.2)	99 (3.0)	5 (0.1)	1049 (31.4)	201 (6.0)	304 (9.1)
Spain	3639 (21.8)	730 (21.8)	679 (20.3)	1599 (47.8)	251 (7.5)	333 (10.0)	1407 (42.1)	868 (25.9)	846 (25.3)
Italy	1992 (11.9)	446 (13.3)	268 (8)	631 (18.9)	111 (3.3)	66 (2.0)	706 (21.1)	987 (29.5)	53 (1.6)
France	551 (3.3)	89 (2.7)	123 (3.7)	47 (1.4)	203 (6.1)	146 (4.4)	20 (0.6)	58 (1.7)	174 (5.2)
United Kingdom	1076 (6.4)	325 (9.7)	101 (3)	212 (6.3)	176 (5.3)	437 (13.1)	12 (0.4)	86 (2.6)	412 (12.3)
the Netherlands	1356 (8.1)	366 (10.9)	91 (2.7)	113 (3.4)	286 (8.5)	759 (22.7)	4 (0.1)	109 (3.3)	435 (13.0)
Germany	1995 (11.9)	303 (9.1)	381 (11.4)	132 (3.9)	657 (19.6)	407 (12.2)	36 (1.1)	166 (5.0)	540 (16.1)
Denmark	2005 (12.0)	488 (14.6)	162 (4.8)	204 (6.1)	466 (13.9)	539 (16.1)	27 (0.8)	306 (9.1)	214 (6.4)
Sweden	2915 (17.4)	585 (17.5)	610 (18.2)	166 (5.0)	1097 (32.8)	654 (19.5)	85 (2.5)	565 (16.9)	368 (11.0)

BMI indicates body mass index; EPIC‐CVD, European Prospective Investigation into Cancer and Nutrition–Cardiovascular Disease; MUFAs, monounsaturated fatty acids; PUFAs, polyunsaturated fatty acids; and SFAs, saturated fatty acids.

* All values are number (percent) or median (interquartile range).

**Table 2:** Baseline Dietary Characteristics in the Lowest (Q1) and Highest (Q5) Quintiles of Dietary Fatty Acid Intake (in Percent of Energy Intake) in the EPIC‐CVD Case‐Cohort Study Subcohort (n=16 730)

Dietary Factors	All Subcohort, n=16 730	Total Fatty Acids		SFAs		MUFAs		PUFAs	
		Q1, n=3346	Q5, n=3346	Q1, n=3346	Q5, n=3346	Q1, n=3346	Q5, n=3346	Q1, n=3346	Q5, n=3346
Total energy intake, kcal	1904 (1551–2343)	1754 (1423–2153)	2015 (1630–2474)	1789 (1447–2202)	2031 (1647–2474)	1738 (1417–2105)	1962 (1591–2420)	1897 (1512–2345)	1890 (1545–2327)
Fatty acids, %TEI									
Total	35.0 (30.9–39.2)	27.4 (25.1–28.8)	43.2 (41.5–45.7)	29.3 (25.6–33.6)	40.0 (37.6–43.0)	28.5 (25.8–30.6)	40.9 (37.7–44.7)	30.9 (27.3–35.2)	37.8 (34.2–41.7)
SFAs	14.0 (11.7–16.4)	10.9 (9.1–12.5)	16.7 (13.8–19.5)	9.7 (8.5–10.5)	18.7 (17.8–20.1)	12.4 (10.6–14.1)	13.0 (11.2–15.2)	12.9 (10.6–15.5)	14.0 (11.8–16.1)
MUFAs	14.1 (11.9–17.0)	10.4 (9.3–12.1)	18.9 (16.1–23.8)	14.2 (10.3–17.9)	14.8 (13.4–16.5)	10.2 (9.2–10.8)	21.1 (19.3–24.0)	13.9 (11.1–16.8)	13.9 (12.0–16.3)
PUFAs	5.5 (4.5–7.0)	4.6 (3.9–5.7)	6.3 (5.1–8.3)	5.1 (4.2–6.8)	5.7 (4.7–6.9)	5.4 (4.2–6.8)	5.2 (4.5–6.4)	3.9 (3.6–4.1)	8.9 (8.1–10.3)
MUFA:SFA ratio	0.9 (0.8–1.3)	0.9 (0.8–1.3)	1.1 (0.8–1.7)	1.5 (1.1–2.0)	0.8 (0.7–0.9)	0.8 (0.7–0.9)	1.7 (1.4–2.0)	1.1 (0.8–1.4)	1.0 (0.9–1.2)
PUFA:SFA ratio	0.4 (0.3–0.5)	0.4 (0.3–0.6)	0.4 (0.3–0.5)	0.6 (0.4–0.7)	0.3 (0.2–0.4)	0.4 (0.3–0.6)	0.4 (0.3–0.5)	0.3 (0.2–0.4)	0.7 (0.5–0.8)
Protein, %TEI									
Total	18.0 (15.9–20.3)	18.1 (15.9–20.6)	17.3 (15.5–19.8)	19.1 (16.5–21.7)	17.1 (15.1–19.3)	17.8 (15.7–20.2)	18.5 (16.3–21.0)	17.8 (15.8–20.0)	18.0 (15.9–20.5)
Plant origin	5.4 (4.5–6.4)	6.2 (5.3–7.3)	5.0 (3.9–6.0)	6.8 (6.0–7.7)	4.3 (3.6–5.0)	5.8 (5.0–6.7)	5.8 (4.9–6.7)	5.6 (4.7–6.6)	5.5 (4.6–6.5)
Animal origin	11.1 (8.8–13.6)	10.4 (8.1–13.1)	11.1 (9–13.7)	11.1 (8.5–14.1)	11.1 (8.9–13.5)	10.3 (8.0–12.9)	11.7 (9.5–14.4)	10.8 (8.6–13.3)	10.9 (8.7–13.6)
Mixed origin	1.2 (0.6–2.0)	1.2 (0.6–1.9)	0.9 (0.4–1.7)	0.8 (0.3–1.4)	1.4 (0.8–2.2)	1.4 (0.8–2.1)	0.6 (0.3–1.2)	1.1 (0.5–1.8)	1.3 (0.6–2.1)
Carbohydrate, %TEI)	46.6 (42.1–51.1)	54.7 (51.9–57.7)	38.6 (35.7–41.4)	50.9 (45.9–55.8)	42.6 (38.9–45.8)	53.6 (50.4–57.0)	40.1 (36.5–43.6)	50.7 (46.3–55.0)	43.8 (39.6–47.7)
GI	56.0 (53.6–58.5)	56.5 (53.8–59.2)	55.2 (52.7–57.6)	57.0 (53.9–59.8)	55.7 (53.3–58.0)	56.0 (53.5–58.5)	55.3 (52.9–57.7)	56.2 (53.6–59.0)	55.8 (53.4–58.4)
Dietary fiber, g/d	21.8 (17.3–27.2)	23.6 (18.8–29.3)	20.1 (15.9–25.1)	24.2 (19.2–30.5)	19.4 (15.2–24.5)	23.1 (18.6–28.6)	21.8 (17.5–27.1)	21.4 (17.0–26.8)	22.0 (17.3–27.3)
Fruits and vegetables, g/d	396.9 (249.0–594.2)	436.8 (283.4–620.1)	448.8 (245.1–725.3)	542.6 (370.3–750.8)	275.7 (182.1–416.0)	392.5 (255.7–561.2)	602.7 (421.9–815.4)	435.5 (273.1–635.9)	396.6 (251.9–600.1)
Total dairy	275.7 (159.1–441.4)	299.7 (161.2–488.7)	239.7 (134.2–368.9)	213.4 (98.5–348.3)	333.3 (204.4–527.1)	358.2 (210.6–556.9)	214.8 (119.5–325.4)	302.1 (180.9–476.4)	252.4 (139.8–394.2)
Milk	155.3 (32.8–293)	178.6 (37.5–342.7)	109.3 (19.3–227.7)	150 (21.7–256.1)	156.3 (36.1–339.3)	200.0 (49.9–400.0)	121.4 (22.3–225.2)	176.4 (53.5–320.0)	139.5 (22.1–277.6)
Yogurt/thick fermented milk	26.3 (0–93.3)	35.7 (0.0–113.0)	17.9 (0.0–57.4)	8.3 (0.0–55.4)	35.7 (2.5–107.1)	53.8 (8.8–125.0)	13.4 (0.0–42.9)	21.4 (0.0–107.1)	21.4 (0.0–71.4)
Cheese	30 (14.6–54.2)	20.3 (8.1–37.6)	44.3 (22.1–77.1)	14.2 (2.6–29.2)	42.9 (23.2–75.5)	22.3 (10.9–40.1)	38.1 (13.5–70.8)	35.7 (15.9–62.6)	25.1 (11.4–45.4)
Added fats	29 (18.6–41.7)	18.3 (11.3–27.3)	44.5 (31.4–59.1)	26.9 (16.6–38.0)	33.6 (23.3–47.9)	18.3 (11.1–27.9)	42.4 (32.0–54.9)	24.6 (15.8–35.2)	33.1 (23.1–47.5)
Vegetable oils	6.8 (1.3–25.9)	4.6 (0.7–16.2)	24.2 (3.4–46.4)	23.1 (8.4–34.7)	2.6 (0.2–6.7)	2.3 (0.2–5.7)	38.6 (28.2–50.4)	15.7 (1.3–27.9)	7.9 (2.9–26.0)
Butter	0.0 (0.0–2.2)	0 (0.0–1.3)	0.0 (0.0–2.0)	0.0 (0.0–0.2)	1.4 (0.0–14.3)	0.2 (0.0–2.6)	0.0 (0.0–0.5)	0.1 (0.0–2.2)	0.0 (0.0–2.0)
Margarine	4 (0.1–20.6)	3.2 (0.0–12.8)	2.3 (0.0–20.8)	0.1 (0.0–3.7)	13.3 (1.2–34.9)	8.6 (1.3–20.0)	0.2 (0.0–2.0)	0.3 (0.0–4.2)	10.4 (0.3–26.0)
Meat	100.7 (67.6–140.3)	82.0 (54.1–115.4)	106.3 (72.0–153.3)	93.7 (60.3–134.2)	103.3 (71.4–144.6)	78.1 (50.3–112.0)	104.2 (70.6–151.4)	86.1 (57.2–121.9)	105.9 (68.7–148.9)
Red meat	39.3 (19.6–65.5)	30.8 (12.9–53.8)	44.1 (24.0–68.3)	33.7 (16.1–56.5)	38.1 (18.6–66.0)	31.5 (13.4–55.5)	45.1 (25.9–68.7)	33.4 (15.3–57.6)	39.3 (20.7–65.6)
Processed meat	25.9 (12–47.5)	18.7 (9.0–33.4)	25.2 (5.0–55.8)	17.4 (6.5–35.1)	35.1 (19.0–60.3)	18.6 (9.0–32.9)	16.3 (2.6–39.6)	19.8 (8.6–36.2)	27.4 (11.1–52.2)
Poultry	16.1 (7.2–31.1)	15.6 (7.0–30.0)	18.3 (7.1–35.4)	23.7 (11.4–42.5)	10.3 (3.3–20.8)	11.1 (4.2–23.2)	24.4 (13.0–39.3)	15.7 (7.2–29.1)	16.4 (6.7–35.6)
Cakes and biscuits	27.9 (10.7–55.8)	21.4 (7.5–42.9)	25.4 (9.8–52.7)	14.1 (1.1–32.5)	37.4 (15.8–69.0)	24.5 (10.5–46.8)	19.2 (5.3–42.9)	26.1 (8.9–56.4)	25.9 (9.9–51.1)
Sugar and confectionary	29.5 (14.9–52.4)	29.3 (13.7–52.9)	23.1 (11.5–43.4)	20.0 (9.2–36.4)	36.2 (18.5–62.0)	33.3 (16.2–57.5)	20.0 (10.0–34.1)	31.5 (15.5–54.6)	24.4 (12.3–45.6)
Cereal and cereal products	197.0 (140.4–273.0)	216.2 (148.6–309.8)	172.7 (124.8–230.6)	228.7 (157.2–323.7)	170.4 (124.3–231.7)	195.9 (137.7–269.2)	194.7 (140.0–263.3)	218.2 (147.9–320.6)	184.6 (132.5–249.3)
Eggs and egg products	14.3 (6.9–24.8)	9.8 (3.6–21.0)	15.2 (7.4–27.7)	14.2 (6.1–26.0)	14.5 (6.6–25.6)	9.6 (3.6–20.8)	15.9 (8.0–28.4)	11.2 (4.4–20.6)	16.1 (8.2–27.4)
Condiments and sauces	13.2 (6.3–25.2)	10.1 (4.5–19.9)	16.2 (7.6–30.6)	10.5 (4.5–18.8)	16.4 (7.6–33.0)	11.4 (4.9–22.6)	11.4 (5.5–19.6)	8.3 (3.9–16.9)	17.8 (8.5–33.0)
Fish and shellfish	28.3 (14.7–50.6)	25.1 (11.8–47.3)	27.3 (14.8–49.8)	38.1 (18.5–69.0)	23.8 (12.3–41.7)	19.3 (8.8–39.2)	33 (18.7–62.4)	24.2 (12.5–43.8)	30.3 (14.2–55.4)
Nuts and seeds	0.6 (0–3.4)	0.3 (0.0–1.6)	1.3 (0.0–5.3)	0.2 (0.0–2.1)	0.7 (0.0–3.4)	0.7 (0.0–2.3)	0.3 (0.0–5.3)	0.2 (0.0–0.8)	1.6 (0.0–7.1)

All values are median (interquartile range). All food intakes are expressed as grams per day. %TEI indicates percentage of total energy intake; EPIC‐CVD, European Prospective Investigation into Cancer and Nutrition–Cardiovascular Disease; GI, glycemic index; MUFAs, monounsaturated fatty acids; PUFAs, polyunsaturated fatty acids; and SFAs, saturated fatty acids.

Dairy products, particularly cheese, contributed most to dietary SFAs in all countries (Table S3), whereas food sources of unsaturated fatty acids were diverse. For instance, vegetable oils contributed over 45% to dietary MUFAs in Southern European countries but <1% in Northern Europe, where meat was the predominant source of MUFAs (20.4%–31.4%) (results not shown). Positive correlations of foods with fatty acids reflected their fatty acid composition and contributions to fatty acid intakes (Figure S3), except the inverse correlation of total dairy with MUFAs, despite dairy being within the top 3 MUFA food sources in all EPIC countries. Inverse correlations were observed for cereal products (for SFAs and MUFAs), fruit and vegetables (SFAs), and butter (PUFAs).

### Fatty Acids and CHD, and Effect Modification

**Figure 1:**
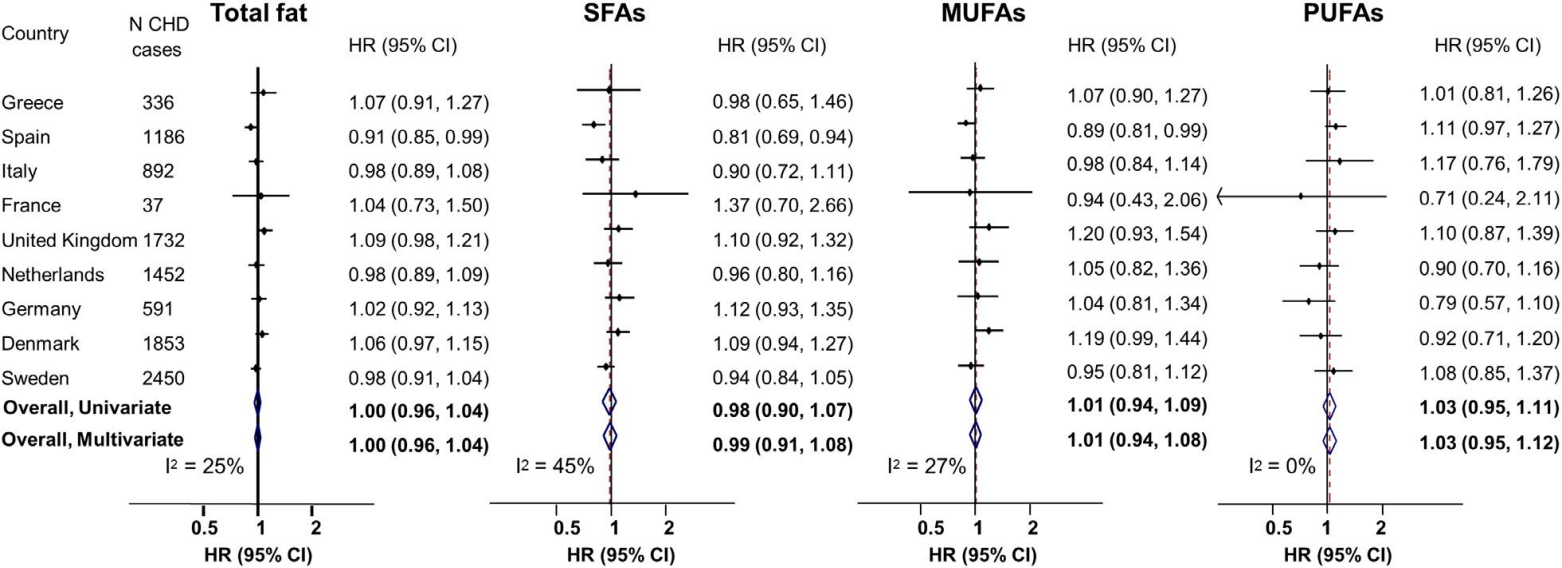
Associations of dietary consumption of each of total fat, saturated fatty acids (SFAs), monounsaturated fatty acids (MUFAs), and polyunsaturated fatty acids (PUFAs) (all per 5% of total energy intake) with incidence of coronary heart disease (CHD) in the EPIC‐CVD (European Prospective Investigation into Cancer and Nutrition–Cardiovascular Disease) study. Hazard ratios (HRs) and 95% CIs for each 5% higher energy intake from total fat, SFAs, MUFAs, and PUFAs were analyzed within each country separately, with age as the underlying time variable and the baseline hazard stratified by sex. The multivariable‐adjusted HR included adjustment for age at recruitment (years), center, energy intake (kcal/d), education (low, medium, high), smoking (never, former, current), physical activity (inactive, moderately inactive, moderately active, active), alcohol intake (0, 0–6, 6–12, 12–24, >24 g/d), dietary fiber (g/d, continuous), fruit and vegetable consumption (g/d, continuous), body mass index (kg/m^2^, continuous), preexisting diabetes mellitus, hypertension, and hyperlipidemia. Country‐specific HRs (95% CIs) were combined in univariate and multivariate random‐effects meta‐analyses to obtain pooled HRs and 95% CIs.

**Figure 2:**
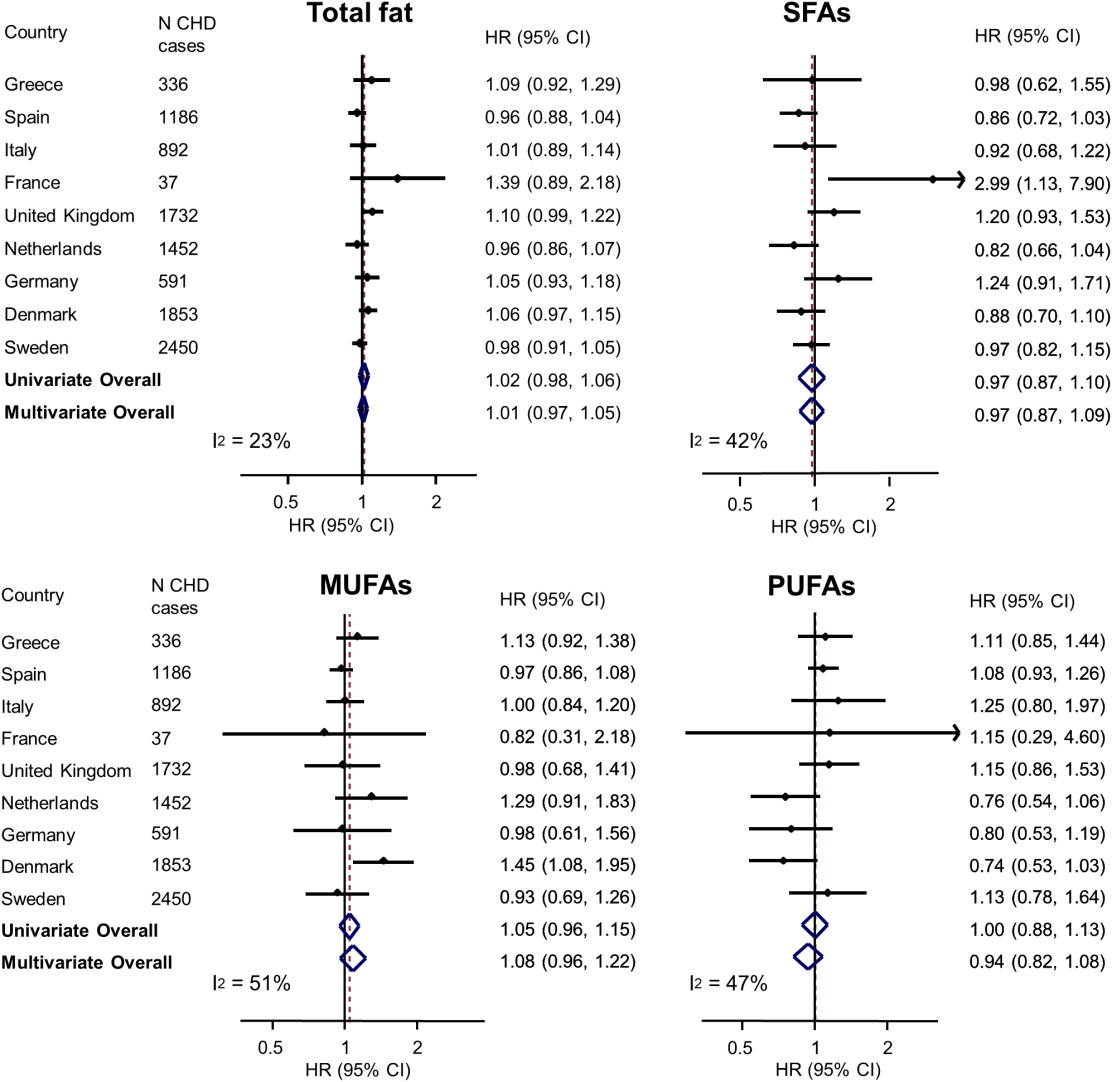
The associations with coronary heart disease (CHD) of substituting 5% energy intake from dietary fatty acids (total and classes) for 5% energy from carbohydrates in the EPIC‐CVD (European Prospective Investigation into Cancer and Nutrition–Cardiovascular Disease) study. Hazard ratios (HRs) and 95% CIs for each 5% higher energy intake from total fatty acids, saturated fatty acids (SFAs), monounsaturated fatty acids (MUFAs), and polyunsaturated fatty acids (PUFAs), to substitute for 5% lower energy intake from carbohydrates, were analyzed within each country separately, with age as the underlying time variable and the baseline hazard stratified by sex. The multivariable‐adjusted HR included adjustment for age at recruitment (years), center, energy intake (kcal/d), smoking (never, former, current), education (low, medium, high), physical activity (inactive, moderately inactive, moderately active, active), alcohol intake (0, 0–6, 6–12, 12–24, >24 g/d), dietary fiber, fruit, and vegetable consumption, body mass index, preexisting diabetes mellitus, hypertension and hyperlipidemia, and all macronutrients except the replacement nutrient (carbohydrates) (i.e., animal‐derived protein, plant‐derived protein, and mixed‐origin protein), and total fat or, for specific fatty acid analysis, SFAs, MUFAs, and PUFAs. Country‐specific HRs (95% CIs) were combined in univariate and multivariate random‐effects meta‐analyses to obtain pooled‐effect estimates and 95% CIs.

**Figure 3:**
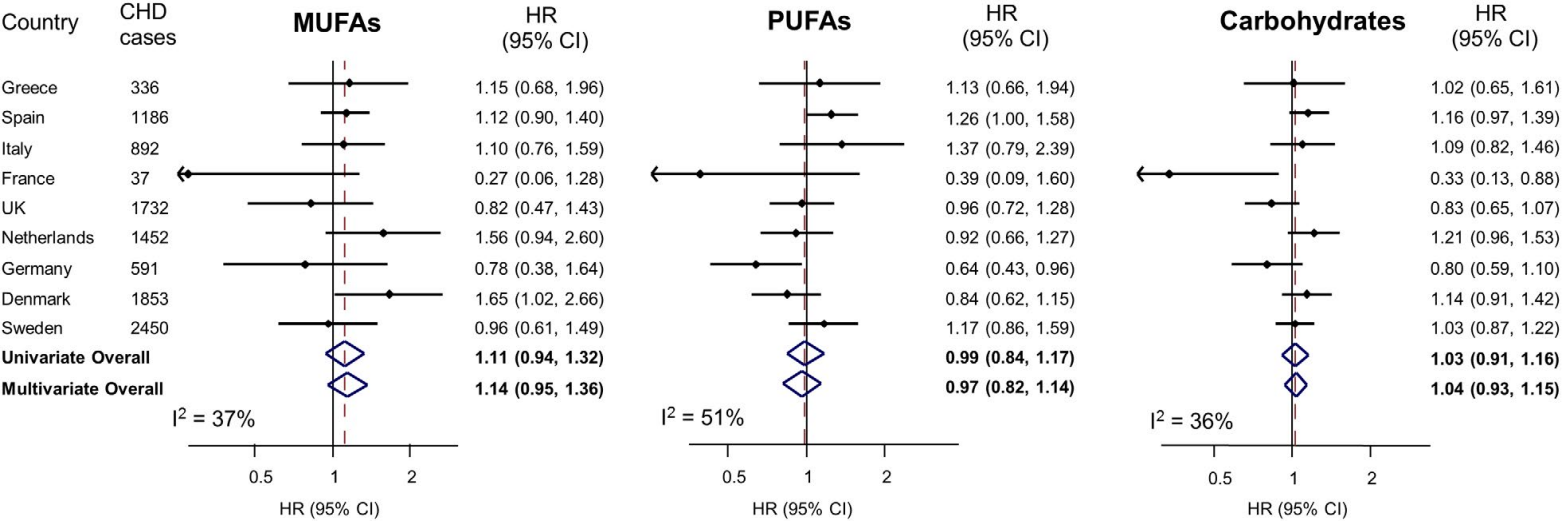
The associations with coronary heart disease (CHD) of substituting 5% energy intake from monounsaturated fatty acids (MUFAs), polyunsaturated fatty acids (PUFAs), or carbohydrates for 5% energy from saturated fatty acids (SFAs) in the EPIC‐CVD (European Prospective Investigation into Cancer and Nutrition–Cardiovascular Disease) study. Hazard ratios (HRs) and 95% CIs for each 5% higher energy intake from MUFAs, PUFAs and carbohydrates, to substitute for 5% lower energy intake from SFAs, were analyzed within each country separately, with age as the underlying time variable and the baseline hazard stratified by sex. The multivariable‐adjusted HR included adjustment for age at recruitment (years), center, energy intake (kcal/d), education (low, medium, high), smoking (never, former, current), physical activity (inactive, moderately inactive, moderately active, active), alcohol intake (0, 0–6, 6–12, 12–24, >24 g/d), dietary fiber, fruit and vegetable consumption, body mass index, preexisting diabetes mellitus, hypertension, and hyperlipidemia, and all macronutrients except the replacement nutrient (SFAs) (i.e., MUFAs, PUFAs, carbohydrates, animal‐derived protein, plant‐derived protein, and mixed‐origin protein). Country‐specific HRs (95% CIs) were combined in univariate and multivariate random‐effects meta‐analysis to obtain pooled effect estimates and 95% CIs.

Total fatty acids, SFAs, MUFAs, or PUFAs were not associated with incident CHD (Figure 1, Table S5, Figures S4 and S5). Substituting energy from total fatty acids, SFAs, MUFAs, or PUFAs for energy from carbohydrates was not associated with CHD (Figure 2). Similarly, substituting energy from MUFAs, PUFAs, or carbohydrates for energy from SFAs was not associated with CHD (Figure 3). There were some differences in associations among countries. For instance, substituting SFAs for carbohydrates was associated with a higher CHD incidence in France (HR, 2.99; 95% CI, 1.13–7.90), whereas there was no association in any other country or overall (pooled HR, 0.97; 95% CI, 0.87–1.09; *I* ^2^=42%; Figure 2). There was no evidence from an interaction test that the association varied by GI levels of diet (*P* for interaction=0.579; Table S6). There was no evidence for differences in associations between men and women, between different age groups, or by plausibility of energy reporting or by preexisting diabetes mellitus, hypertension, and hyperlipidemia (Tables S7 and S8).

### Food Sources of SFAs and CHD

Each 1%TEI from SFAs from yogurt, cheese, and fish was associated with a 7% (HR, 0.93; 95% CI, 0.88–0.99; *P*=0.017), 2% (HR, 0.98; 95% CI, 0.96–1.00; *P*=0.018), and 13% (HR, 0.87; 95% CI, 0.75–1.00; *P*=0.048) lower CHD incidence, respectively (Table 3). In contrast, SFAs from red meat and butter, respectively, were associated with a 7% (HR, 1.07; 95% CI, 1.02–1.12; *P*=0.007) and 2% (HR, 1.02; 95% CI, 1.00–1.04; *P*=0.032) higher CHD incidence per 1%TEI. After adjusting for SFAs from other food sources, these HRs remained similar. Specific macronutrient substitutions for SFAs from food sources reflected these findings, including higher CHD incidence when substituting carbohydrates for SFAs from fermented dairy products (yogurt and cheese) and lower CHD incidence when substituting specific macronutrients for SFAs from red meat (Table S9).

**Table 3:** Associations With Coronary Heart Disease of Dietary SFAs From Different Food Sources (per 1%TEI), Without and With Adjustment for SFAs From Any Other Foods in the EPIC‐CVD Case‐Cohort Study

SFAs From Food Source	Contribution of Food to SFAs, %TEI*	Multivariable Adjustments^†^			Additional Adjustment for SFAs From Any Other Food Sources^‡^		
		HR (95% CI) per 1%TEI	*P* Value	*I* ^2^	HR (95% CI) per 1%TEI	*P* Value	*I* ^2^
Dairy products	4.4 (2.9–6.2)	0.98 (0.97–1.00)	0.027	0	0.99 (0.97–1.00)	0.060	1
Milk	0.7 (0.1–1.6)	1.01 (0.99–1.04)	0.239	0	1.01 (0.98–1.04)	0.464	0
Yogurt/thick fermented milk	0.1 (0.0–0.5)	0.93 (0.88–0.99)	0.017	0	0.93 (0.88–0.99)	0.016	0
Cheese	2.3 (1.2–4.0)	0.98 (0.96–1.00)	0.018	10	0.98 (0.96–0.99)	0.007	6
Added fats	2.7 (1.7–4.1)	1.02 (1.00–1.04)	0.018	23	1.02 (1.00–1.03)	0.061	5
Vegetable oils	0.5 (0.1–1.7)	1.03 (0.96–1.11)	0.426	0	1.01 (0.94–1.09)	0.804	0
Butter	0.0 (0.0–0.5)	1.02 (1.00–1.04)	0.032	4	1.02 (1.00–1.04)	0.058	3
Margarine	0.4 (0.0–1.9)	1.00 (0.97–1.03)	0.979	28	1.00 (0.98–1.03)	0.885	15
Meat	2.2 (1.4–3.2)	1.04 (1.01–1.08)	0.013	35	1.05 (1.00–1.09)	0.053	54
Red and processed	1.8 (1.1–2.9)	1.05 (1.01–1.09)	0.015	43	1.05 (1.00–1.10)	0.048	57
Red	0.7 (0.04–1.3)	1.07 (1.02–1.12)	0.007	15	1.07 (1.01–1.12)	0.020	26
Processed	0.8 (0.3–1.6)	1.03 (0.99–1.07)	0.150	16	1.04 (0.98–1.09)	0.196	44
Poultry	0.1 (0.1–0.3)	0.95 (0.80–1.13)	0.552	23	0.93 (0.80–1.08)	0.360	10
Cakes and biscuits	0.9 (0.4–1.8)	0.97 (0.94–1.00)	0.069	8	0.98 (0.94–1.01)	0.210	32
Sugar and confectionary	0.4 (0.1–0.9)	1.00 (0.96–1.03)	0.816	0	0.99 (0.96–1.03)	0.748	0
Cereal and cereal products	0.4 (0.3–0.6)	1.08 (0.93–1.25)	0.333	41	1.08 (0.93–1.25)	0.329	40
Egg and egg products	0.2 (0.1–0.4)	0.90 (0.79–1.04)	0.143	0	0.90 (0.78–1.03)	0.121	0
Condiments and sauces	0.2 (0.1–0.4)	0.97 (0.82–1.15)	0.725	54	0.95 (0.81–1.13)	0.596	52
Fish and shellfish	0.1 (0.1–0.3)	0.87 (0.75–1.00)	0.048	0	0.85 (0.74–0.99)	0.031	0
Nuts and seeds	0.0 (0.0–0.1)	0.82 (0.65–1.03)	0.089	36	0.83 (0.65–1.05)	0.111	37

%TEI indicates percentage of total energy intake; EPIC‐CVD, European Prospective Investigation into Cancer and Nutrition–Cardiovascular Disease; HR, hazard ratio; and SFAs, saturated fatty acids.

* All values are median (interquartile range) in the overall subcohort (n=16 730).

^†^ HRs and 95% CIs for each 1% higher energy intake from SFAs from each food group were analyzed within each country separately, with age as the underlying time variable and the baseline hazard stratified by sex. The multivariable‐adjusted HR included adjustment for age at recruitment (years), center, energy intake (kcal/d), education (low, medium, high), smoking (never, former, current), physical activity (inactive, moderately inactive, moderately active, active), alcohol intake (0, 0–6, 6–12, 12–24, >24 g/d) dietary fiber (g/d, continuous), fruit and vegetable consumption (g/d, continuous), body mass index (kg/m^2^, continuous), preexisting diabetes mellitus, hypertension, and hyperlipidemia. Country‐specific HRs (95% CIs) were combined in a multivariate random‐effects meta‐analysis to obtain pooled‐effect estimates and 95% CIs. The analysis included all 16 730 subcohort members and 10 529 coronary heart disease cases.

^‡^ Additionally adjusted for the sum of SFAs (%TEI) from all other food sources.

### Ancillary Analyses

There were some geographical differences in associations of SFAs (*P*_heterogeneity_=0.029) and of PUFAs substituting for SFAs (*P*_heterogeneity_=0.039), but none of the region‐specific estimates were significant (eg, the HR for substituting PUFAs for SFAs was 0.84 [95% CI, 0.69–1.02] in Central Europe versus 1.17 [95% CI, 0.93–1.47] in the South) (Table S10). Investigating fatal or nonfatal CHD separately, excluding the first 2 years of follow‐up, excluding extreme energy reporters, Winsorizing covariates, and 1‐stage analysis did not materially change the results (Tables S11 and S12). Regression calibration analyses with 24‐hour recall data made no difference to our findings (results not shown).

## Discussion

In this large pan‐European observational study, total dietary fatty acids and their classes (SFAs, MUFAs, and PUFAs) were not associated with CHD, regardless of which other macronutrient was substituted for. By contrast, we observed directionally opposite associations of SFAs with CHD incidence depending on the food sources. For example, whereas consuming SFAs from fermented dairy products and fish was associated with lower CHD incidence, intake of SFAs from red meat and butter was associated with higher CHD incidence. These results suggest the importance of the overall food matrix alongside the consideration of the nutrient composition.

### Findings in Context of Prior Evidence

To our knowledge, our study is the first to report on associations of dietary fatty acids and incident CHD across multiple regions in Europe with diverse diets,^27^ allowing standardized investigation of various intake levels and food sources. Overall, SFA intake was high, with <12% of participants consuming less than the recommended upper limit of 10%TEI from SFAs.^2^ Consistent with most previous evidence,^5^, ^37^, ^38^ we observed essentially null associations of dietary fatty acids with CHD incidence in a random‐effects meta‐analysis when not considering the substitution nutrient. Our observation of null CHD association when substituting PUFAs for carbohydrates was consistent with results from the global PURE (Prospective Urban Rural Epidemiology) study, which involved participants from 18 countries in 5 continents.^14^ However, our observed null association when substituting PUFAs for SFAs differed from studies of CHD based in the United States and Northern Europe reporting inverse associations.^8^, ^9^, ^10^, ^23^ This is in line with mixed findings from other studies in Europe, including a positive association with composite CVD in the UK Biobank study (with 10 724 CVD cases),^15^ and an inverse association with composite CVD among Spanish adults from the PREDIMED (Prevención con Dieta Mediterránea) study (with 336 CVD cases).^16^ The latter results are not directly comparable to our findings, because these were based on an observational follow‐up of a randomized clinical trial among those at high CVD risk (PREDIMED study),^16^ whereas our study was based in a general population. Because associations in UK Biobank and the PREDIMED study were with composite CVD only, it is unclear to what extent these are driven by CHD or other CVD subtypes such as stroke. Importantly, our results are consistent with a previous Dutch cohort reporting a null CHD association of substituting PUFAs for SFAs.^12^

Some meta‐analyses of randomized trials,^4^, ^5^ but not all,^6^ have reported a beneficial effect on CVD or CHD risk of substituting PUFAs for SFAs. In addition to these heterogeneous findings across trials, the types and quantities of SFA‐rich foods consumed in trial participants may differ from those in free‐living populations.^17^ The potential inverse association with CHD of substituting PUFAs for SFAs from butter, but not fermented dairy, highlights the importance of considering the food source of the SFAs.

The relevance of substituting MUFAs for SFAs to CHD risk is largely unaccounted for in trials,^2^ whereas observational studies of CHD have variously reported inverse,^23^ null,^12^ and positive^9^, ^10^, ^11^ associations, potentially because of differences between plant‐ and animal‐derived MUFAs.^22^ Although substituting MUFAs for SFAs was associated with lower composite CVD incidence in the PREDIMED study,^16^ there was no strong evidence for an association in UK Biobank (*P*=0.21).^15^ In the present study, substituting MUFAs for SFAs was not associated with CHD. Data on plant‐ versus animal‐derived MUFAs were not available. However, associations were consistently null across all European regions we studied, regardless of food sources being mainly plant based (vegetable oils) in Southern European centers and mainly animal based (dairy and meat) in the other centers.

Previous studies attempted to test whether a diet with low carbohydrate quality may weaken the association of SFA intakes with CHD risk.^12^, ^23^, ^24^, ^25^ However, we found no evidence for the potential effect modification caused by carbohydrate quality assessed by GI. The lack of effect modification could reflect multiple factors. Our GI measure for the multiple European populations was limited, because true GI values may have varied substantially among populations.^39^ Moreover, GI as a marker of carbohydrate quality is limited, because it captures the effect of glucose only, not other monosaccharides, and the effects of GI cannot be separated from fiber and sugar intake that vary by low‐ or high‐GI foods. Further research is warranted to assess carbohydrate quality and the interaction with fat quality, for example, by using biomarkers for sugar intake. Potential nutritional biomarkers in urine or blood have been identified, and these can offer more precise objective assessment to complement the information from subjective dietary assessment for sugar consumption.^40^ There is also a need to improve databases of sugar contents and GI values of diverse food products in diverse settings.^39^

Previous observational studies investigating SFAs from food sources with CHD are limited with inconsistent results. Specifically, the inverse association of SFAs from dairy, driven by yogurt and cheese in EPIC‐CVD, is consistent with 2 smaller studies that included 1807^11^ and 316^18^ CHD cases, but not with other large studies reporting null associations.^12^, ^13^, ^19^, ^20^ Similarly, the higher CHD incidence associated with SFAs from red and total meat is consistent with some,^13^, ^18^ but not all studies.^11^, ^12^, ^13^ Observational evidence of macronutrient‐specific substitutions for fatty acids from different foods in relation to CHD is scarce, and focused on fats from total dairy and/or meat.^19^, ^21^ By contrast, our study modeled several specific macronutrient substitutions for SFAs from fermented dairy, butter, and red meat, finding opposite associations with CHD depending on SFA food sources and the substitution macronutrient.

### Potential Explanations

The observed opposing associations of lower CHD incidence with SFAs from fermented dairy, and of higher CHD incidence with SFAs from red meat, may cancel each other out to create a null association for total SFA intake, highlighting the importance of considering the food sources of nutrients. A key mechanism proposed to link SFAs to increased CHD risk involves effects of dietary fatty acids on lipid metabolism, including low‐density lipoprotein–raising effects of dietary SFAs.^1^, ^7^ It is possible that different food sources of fatty acids contributed to a lack of differences in non–HDL‐C across total SFA intake levels, because meat and dairy consumption have opposite associations (positive and inverse, respectively) with plasma non–HDL‐C in EPIC‐CVD.^41^ Although we cannot establish causality from these observational findings, consuming total fat and SFAs from cheese raised circulating low‐density lipoprotein cholesterol concentrations less than consuming similar amounts of fatty acids from butter in trials,^42^, ^43^ and the effects of red meat consumption on circulating lipids depend on the comparison diet,^44^ also pointing to the importance of the food matrix.

Specific SFA isomer composition of different foods may contribute to their associations with CHD.^11^, ^45^ For instance, observational evidence suggests that odd‐chain and even‐chain SFAs are associated differently with CHD incidence,^37^ and trial evidence indicates different directions of effect of different individual SFAs on serum lipids.^7^ Thus, food‐specific associations with CHD may be attributable to the different mix of individual SFA isomers and other fatty acids in addition to other nutrients and bio‐active components in foods or by correlations with other foods or behaviors. Vitamin K, bioactive peptides generated during fermentation, and probiotics in yogurt may contribute to inverse associations of SFAs from fermented dairy products with CHD.^45^ Equally, dietary SFAs, iron, phosphatidylcholine, L‐carnitine, and advanced glycation end products may all contribute to mechanisms underlying positive associations of red meat, and hence SFAs from red meat in the current study, with CHD.^46^ Although we have observed food‐specific associations of SFAs with CHD, it is impossible to confirm within an observational study whether associations are specific to SFAs versus other constituents of those foods. Our finding of an inverse association of SFAs from fish, which contributes more to dietary PUFAs than to SFAs, further highlights the limited ability to separate potential effects of different nutrients within foods or to attribute causality specifically to SFAs from observed associations.

### Strengths and Limitations

Strengths of the current study include the large sample size involving participants from 9 European countries with heterogeneous dietary habits^27^ and comprehensive analyses addressing macronutrient‐specific substitutions, potential effect modification by carbohydrate quality (GI), and associations of SFAs from different food sources.

The study’s potential limitations merit consideration. Our principal findings were not highly significant, suggesting the need for further evaluation of this hypothesis in additional studies and in further populations. No standardized data were available across all EPIC‐CVD countries to reliably investigate associations of n3‐ and n6‐PUFAs, plant‐ versus animal‐derived SFAs, MUFAs, or PUFAs, or of subtypes of carbohydrates (eg, sugar and starch), separately. Nutrient intake from nutritional supplements was not available. Our study included largely White participants, and our findings may therefore not be generalizable to other ethnic/racial groups. Our findings reflect associations among participants in an observational study, and therefore our report is based on self‐reported dietary intakes and food sources of fatty acids in this study population, rather than causal effects of changes in fatty acid intake, which would require an experimental design. Our findings reflect associations within the range of dietary intake levels observed within our study population. It is possible that generalizability to other populations in different countries may be affected by differences in dietary patterns, risk factor profiles, and nutrient contents of food sources of SFAs (eg, fortification with micronutrients if such nutrients are causally related to CHD). Therefore, our findings should be further confirmed in other large‐scale observational studies among diverse populations with different intake levels, and in intervention studies when feasible, acknowledging that the latter may pose major challenges of logistics, cost, and adherence with interventional dietary regimes over the required period of time until end points develop. Despite overall null associations of dietary fatty acids with CHD in our study, there were some country‐specific differences in associations. This may be explained by country‐specific differences in food sources of dietary fatty acids, or residual confounding that might differ among country‐specific study populations. For instance, the positive association of substituting SFAs for carbohydrates in France despite the overall null association in EPIC‐CVD might be explained by specific dietary patterns and unique characteristics of this study population including all‐women education professionals. Nevertheless, there was minimal heterogeneity in associations of SFAs from specific foods with CHD incidence, including of SFAs from dairy, red meat, processed meat, and butter, thus supporting the importance of considering the overall food matrix across all included countries. Despite adjusting for a range of potential confounders, we cannot exclude potential residual confounding from unmeasured factors such as from trans fats. However, trans fat intake has traditionally been lower across Western Europe (mean intake ~1%TEI) compared with North America (~4%TEI).^47^ Moreover, our main findings relate to SFAs from foods containing ruminant trans fats, rather than the harmful industrial trans fats,^38^ making it less likely that our conclusions on the importance of food sources of SFAs would have been materially affected by residual confounding from trans fats. Although we adjusted for self‐reported preexisting hyperlipidemia, information on lipid‐lowering drugs was not available. Hence, if the associations in our study populations were mediated by lipid levels, our findings could underestimate the true associations. Similarly, although we adjusted for self‐reported preexisting hypertension and diabetes mellitus, no information on relevant medication was available. By contrast, our study’s use of single baseline measurements of multiple covariates, potentially with nonrandom measurement error, could have biased associations toward or away from the null, contributing to the overall null associations of fatty acids with CHD. Dietary questionnaires were validated, with estimated validity coefficients for total fat ranging from 0.31 to 0.89 across countries (except Greece: 0.09 in men, 0.26 in women).^30^ This variation among countries may be related to differences in dietary reporting arising from cultural differences or dietary assessment methods as well as center‐specific validation methods. Thus, we cannot exclude the possibility that the country‐specific associations with CHD were influenced by different levels of validity of the measures of exposure among countries. Although our primary results were based on analyses of habitual diet captured by food‐frequency questionnaire data, regression calibration analyses using 24‐hour recall data available among a subset of participants did not change our overall findings or interpretation. We conducted a range of sensitivity analyses, suggesting that the study’s principal results were robust to over‐ and underreporting of energy intake. Our current work focused on food‐specific associations of SFAs only, based on prior evidence of differences in associations with CHD of dairy products^19^, ^20^, ^35^, ^36^ and meat,^35^ 2 major food sources of SFAs. Future studies need to investigate how the overall food matrix affects CHD risk, ideally within causally informative designs.

### Potential Public Health Implications

The observed opposing associations of SFAs from fermented dairy products versus SFAs from red meat suggest that the potential health effects of limiting SFAs in general, and by extension of substituting other macronutrients for SFAs, may be beneficial, neutral, or detrimental (assuming causality) depending on which specific changes in food consumption are made to limit or substitute for SFAs. Overall, shared food sources of SFAs, MUFAs, and PUFAs, their correlations, and food‐specific associations of SFAs in the current study, whether or not causally attributable to fatty acids, emphasize the importance of considering the source foods, rather than macronutrients alone, when evaluating associations with disease risk. Our findings imply that recommendations to limit dietary SFAs and replace them with MUFAs or PUFAs for CHD prevention may not be guaranteed to achieve optimal health benefits without considering the food matrix from which fatty acids are consumed within populations. This study adds to growing evidence suggesting that future dietary guidelines for CHD prevention should consider the totality of evidence on nutrients, foods, and dietary patterns combined.

## Conclusions

This epidemiological study found no strong evidence for associations of total dietary fatty acids, SFAs, MUFAs, and PUFAs with incident CHD, regardless of the substitution nutrients, within the range of intake in this European population. By contrast, we found associations of SFAs with CHD in opposite directions, dependent on the food source, suggesting the importance of the overall food matrix. These observational findings need to be further confirmed but support public health recommendations to consider food sources alongside the individual macronutrients they contain.

## Sources of Funding

EPIC‐CVD was supported by the European Commission Framework Programme 7 (HEALTH‐F2‐2012‐279233), and the European Research Council (268834). The coordinating center was supported by core funding from the: United Kingdom MRC (G0800270; MR/L003120/1), British Heart Foundation (BHF) (SP/09/002; RG13/13/30194; RG/18/13/33946), and National Institute for Health Research (NIHR) Cambridge Biomedical Research Centre (BRC) (BRC‐1215‐20014).* The establishment of the study subcohort was supported by the European Union Sixth Framework Programme (grant LSHM_CT_2006_037197 to the InterAct project) and the MRC Epidemiology Unit (grant MC_UU_00006/1). The coordination of EPIC is financially supported by International Agency for Research on Cancer and also by the Department of Epidemiology and Biostatistics, School of Public Health, Imperial College London, which has additional infrastructure support provided by NIHR Imperial BRC. The national cohorts are supported by: Danish Cancer Society (Denmark); Ligue Contre le Cancer, Institut Gustave Roussy, Mutuelle Générale de l’Education Nationale, Institut National de la Santé et de la Recherche Médicale (France); German Cancer Aid, German Cancer Research Center, German Institute of Human Nutrition Potsdam‐Rehbruecke, Federal Ministry of Education and Research (Germany); Associazione Italiana per la Ricerca sul Cancro‐AIRC‐Italy, Compagnia di SanPaolo and National Research Council (Italy); Dutch Ministry of Public Health, Welfare and Sports, Netherlands Cancer Registry, LK Research Funds, Dutch Prevention Funds, Zorg Onderzoek Nederland, World Cancer Research Fund, Statistics Netherlands (the Netherlands); Health Research Fund– Instituto de Salud Carlos III, Regional Governments of Andalucía, Asturias, Basque Country, Murcia and Navarra, and the Catalan Institute of Oncology (Spain); Swedish Cancer Society, Swedish Research Council and County Councils of Skåne and Västerbotten (Sweden); Cancer Research UK (14136 to EPIC‐Norfolk; C8221/A29017 to EPIC‐Oxford), MRC (1000143 to EPIC‐Norfolk; MR/M012190/1 to EPIC‐Oxford) (United Kingdom). EPIC‐Greece was supported by the Hellenic Health Foundation (Greece). M.S., N.J.W., N.G.F., and F.I. acknowledge support from MRC Epidemiology Unit (MC_UU_00006/1 and MC_UU_00006/3). N.J.W. and N.G.F. acknowledge support from NIHR* Cambridge BRC: Nutrition, Diet, and Lifestyle Research Theme (IS‐BRC‐1215‐20014) and NGF is a NIHR Senior Investigator Award holder. M.S. was also supported by BHF for part of this work while working in the BHF Cardiovascular Epidemiology Unit, Department of Public Health and Primary Care, University of Cambridge, Cambridge, United Kingdom. R.Z.‐R. thanks the “Miguel Servet” program (CP15/00100) from the Institute of Health Carlos III (co‐funded by the European Social Fund–European Social Fund Investing in Your Future). A.W. was supported by a BHF‐Turing Cardiovascular Data Science Award and by the European Commission‐Innovative Medicines Initiative (BigData@Heart). R.C. was funded by a MRC‐Newton project grant to study genetic risk factors of cardiovascular disease among Southeast Asian people and the Academy of Sciences Malaysia (grant no. MR/P013880/1) and a United Kingdom Research and Innovation‐Global Challenges Research Fund Project Grant to study risk factors of noncommunicable diseases in Bangladesh. J.D. holds a BHF Professorship and a NIHR Senior Investigator Award.

The views expressed are those of the author(s) and not necessarily those of the NIHR or the Department of Health and Social Care. Where authors are identified as personnel of the International Agency for Research on Cancer/World Health Organization, the authors alone are responsible for the views expressed in this article and they do not necessarily represent the decisions, policy, or views of the International Agency for Research on Cancer/World Health Organization. The funders of the study had no role in study design, data collection, analysis, data interpretation, or writing of the article, or in the decision to submit for publication. M.S. had full access to all the data in the study, and M.S. and N.G.F. had final responsibility for the decision to submit for publication.

## Disclosures

Dr Danesh reports grants, personal fees and non‐financial support from Merck Sharp & Dohme, grants, personal fees, and nonfinancial support from Novartis, grants from Pfizer, and grants from AstraZeneca outside the submitted work. He is member of the International Cardiovascular and Metabolic Advisory Board for Novartis (since 2010); the Steering Committee of UK Biobank (since 2011); the MRC International Advisory Group (ING) member, London (since 2013); the MRC High Throughput Science ‘Omics Panel Member, London (since 2013); the Scientific Advisory Committee for Sanofi (since 2013); the International Cardiovascular and Metabolism Research and Development Portfolio Committee for Novartis; and the Astra Zeneca Genomics Advisory Board (2018). Dr Butterworth reports grants outside of this work from AstraZeneca, Biogen, BioMarin, Bioverativ, Merck, Novartis, Pfizer, and Sanofi and personal fees from Novartis. The remaining authors have no disclosures to report.

## Supporting information

Tables S1–S12
Figures S1–S5

## References

[1] World Health Organization . Draft guidelines on saturated fatty acid and trans‐fatty acid intake for adults and children. 2018. Available from https://extranet.who.int/dataform/upload/surveys/666752/files/Draft%20WHO%20SFA‐TFA%20guidelines_04052018%20Public%20Consultation(1).pdf. Accessed June 9, 2021. 37490572

[2] Scientific Advisory Committee on Nutrition (SACN) . Saturated fats and health: SACN report 2019. Available from https://www.gov.uk/government/publications/saturated‐fats‐and‐health‐sacn‐report. Accessed June 9, 2021.

[3] Sacks FM , Lichtenstein AH , Wu JHY , Appel LJ , Creager MA , Kris‐Etherton PM , Miller M , Rimm EB , Rudel LL , Robinson JG , et al. Dietary fats and cardiovascular disease: a presidential advisory from the American Heart Association. Circulation. 2017;136:e1–e23. DOI: 10.1161/CIR.0000000000000510. 28620111

[4] Mozaffarian D , Micha R , Wallace S . Effects on coronary heart disease of increasing polyunsaturated fat in place of saturated fat: a systematic review and meta‐analysis of randomized controlled trials. PLoS Med. 2010;7:e1000252. DOI: 10.1371/journal.pmed.1000252. 20351774 PMC2843598

[5] Hooper L , Martin N , Jimoh OF , Kirk C , Foster E , Abdelhamid AS . Reduction in saturated fat intake for cardiovascular disease. Cochrane Database of Systematic Reviews. 2020. DOI: 10.1002/14651858.CD011737.pub3. PMC7388853 32428300

[6] Ramsden CE , Zamora D , Majchrzak‐Hong S , Faurot KR , Broste SK , Frantz RP , Davis JM , Ringel A , Suchindran CM , Hibbeln JR . Re‐evaluation of the traditional diet‐heart hypothesis: analysis of recovered data from Minnesota Coronary Experiment (1968–73). BMJ. 2016;353:i1246. DOI: 10.1136/bmj.i1246. 27071971 PMC4836695

[7] Mensink RP . Effects of Saturated Fatty Acids on Serum Lipids and Lipoproteins: a Systematic Review and Regression Analysis. Geneva: World Health Organization; 2016. Available at: https://apps.who.int/iris/bitstream/handle/10665/246104/9789241565349‐eng.pdf. Accessed June 9, 2021.

[8] Farvid MS , Ding M , Pan A , Sun Q , Chiuve SE , Steffen LM , Willett WC , Hu FB . Dietary linoleic acid and risk of coronary heart disease: a systematic review and meta‐analysis of prospective cohort studies. Circulation. 2014;130:1568–1578. DOI: 10.1161/CIRCULATIONAHA.114.010236. 25161045 PMC4334131

[9] Jakobsen MU , O'Reilly EJ , Heitmann BL , Pereira MA , Balter K , Fraser GE , Goldbourt U , Hallmans G , Knekt P , Liu S , et al. Major types of dietary fat and risk of coronary heart disease: a pooled analysis of 11 cohort studies. Am J Clin Nutr. 2009;89:1425–1432. DOI: 10.3945/ajcn.2008.27124. 19211817 PMC2676998

[10] Virtanen JK , Mursu J , Tuomainen TP , Voutilainen S . Dietary fatty acids and risk of coronary heart disease in men: the Kuopio Ischemic Heart Disease Risk Factor Study. Arterioscler Thromb Vasc Biol. 2014;34:2679–2687. DOI: 10.1161/ATVBAHA.114.304082. 25256234

[11] Praagman J , Beulens JW , Alssema M , Zock PL , Wanders AJ , Sluijs I , van der Schouw YT . The association between dietary saturated fatty acids and ischemic heart disease depends on the type and source of fatty acid in the European Prospective Investigation into Cancer and Nutrition‐Netherlands cohort. Am J Clin Nutr. 2016;103:356–365. 10.3945/ajcn.115.122671. 26791181

[12] Praagman J , de Jonge EA , Kiefte‐de Jong JC , Beulens JW , Sluijs I , Schoufour JD , Hofman A , van der Schouw YT , Franco OH . Dietary saturated fatty acids and coronary heart disease risk in a dutch middle‐aged and elderly population. Arterioscler Thromb Vasc Biol. 2016;36:2011–2018. DOI: 10.1161/ATVBAHA.116.307578. 27417581

[13] Praagman J , Vissers LET , Mulligan AA , Laursen ASD , Beulens JWJ , van der Schouw YT , Wareham NJ , Hansen CP , Khaw KT , Jakobsen MU , et al. Consumption of individual saturated fatty acids and the risk of myocardial infarction in a UK and a Danish cohort. Int J Cardiol. 2019;279:18–26. DOI: 10.1016/j.ijcard.2018.10.064. 30482628 PMC6774776

[14] Dehghan M , Mente A , Zhang X , Swaminathan S , Li W , Mohan V , Iqbal R , Kumar R , Wentzel‐Viljoen E , Rosengren A , et al. Associations of fats and carbohydrate intake with cardiovascular disease and mortality in 18 countries from five continents (PURE): a prospective cohort study. Lancet. 2017;390:2050–2062. DOI: 10.1016/S0140-6736(17)32252-3. 28864332

[15] Ho FK , Gray SR , Welsh P , Petermann‐Rocha F , Foster H , Waddell H , Anderson J , Lyall D , Sattar N , Gill JMR , et al. Associations of fat and carbohydrate intake with cardiovascular disease and mortality: prospective cohort study of UK Biobank participants. BMJ. 2020;368:m688. DOI: 10.1136/bmj.m688. 32188587 PMC7190059

[16] Guasch‐Ferré M , Babio N , Martínez‐González MA , Corella D , Ros E , Martín‐Peláez S , Estruch R , Arós F , Gómez‐Gracia E , Fiol M , et al. Dietary fat intake and risk of cardiovascular disease and all‐cause mortality in a population at high risk of cardiovascular disease. Am J Clin Nutr. 2015;102:1563–1573. DOI: 10.3945/ajcn.115.116046. 26561617

[17] Astrup A , Bertram HCS , Bonjour J‐P , de Groot LCP , de Oliveira Otto MC , Feeney EL , Garg ML , Givens I , Kok FJ , Krauss RM , et al. WHO draft guidelines on dietary saturated and trans fatty acids: time for a new approach? BMJ. 2019;366:l4137. DOI: 10.1136/bmj.l4137. 31270106

[18] de Oliveira Otto MC , Mozaffarian D , Kromhout D , Bertoni AG , Sibley CT , Jacobs DR Jr , Nettleton JA . Dietary intake of saturated fat by food source and incident cardiovascular disease: the Multi‐Ethnic Study of Atherosclerosis. Am J Clin Nutr. 2012;96:397–404. DOI: 10.3945/ajcn.112.037770. 22760560 PMC3396447

[19] Chen M , Li Y , Sun Q , Pan A , Manson JE , Rexrode KM , Willett WC , Rimm EB , Hu FB . Dairy fat and risk of cardiovascular disease in 3 cohorts of US adults. Am J Clin Nutr. 2016;104:1209–1217. DOI: 10.3945/ajcn.116.134460. 27557656 PMC5081717

[20] Dehghan M , Mente A , Rangarajan S , Sheridan P , Mohan V , Iqbal R , Gupta R , Lear S , Wentzel‐Viljoen E , Avezum A , et al. Association of dairy intake with cardiovascular disease and mortality in 21 countries from five continents (PURE): a prospective cohort study. Lancet. 2018;392:2288–2297. DOI: 10.1016/S0140-6736(18)31812-9. 30217460

[21] Vissers LET , Rijksen J , Boer JMA , Verschuren WMM , van der Schouw YT , Sluijs I . Fatty acids from dairy and meat and their association with risk of coronary heart disease. Eur J Nutr. 2019;58:2639–2647. DOI: 10.1007/s00394-018-1811-1. 30167851 PMC6768909

[22] Zong G , Li Y , Sampson L , Dougherty LW , Willett WC , Wanders AJ , Alssema M , Zock PL , Hu FB , Sun Q . Monounsaturated fats from plant and animal sources in relation to risk of coronary heart disease among US men and women. Am J Clin Nutr. 2018;107:445–453. DOI: 10.1093/ajcn/nqx004. 29566185 PMC5875103

[23] Li Y , Hruby A , Bernstein AM , Ley SH , Wang DD , Chiuve SE , Sampson L , Rexrode KM , Rimm EB , Willett WC , et al. Saturated fats compared with unsaturated fats and sources of carbohydrates in relation to risk of coronary heart disease: a prospective cohort study. J Am Coll Cardiol. 2015;66:1538–1548. 10.1016/j.jacc.2015.07.055. 26429077 PMC4593072

[24] Jakobsen MU , Dethlefsen C , Joensen AM , Stegger J , Tjonneland A , Schmidt EB , Overvad K . Intake of carbohydrates compared with intake of saturated fatty acids and risk of myocardial infarction: importance of the glycemic index. Am J Clin Nutr. 2010;91:1764–1768. DOI: 10.3945/ajcn.2009.29099. 20375186

[25] Simila ME , Kontto JP , Mannisto S , Valsta LM , Virtamo J . Glycaemic index, carbohydrate substitution for fat and risk of CHD in men. Br J Nutr. 2013;110:1704–1711. DOI: 10.1017/S0007114513000858. 23534456

[26] Danesh J , Saracci R , Berglund G , Feskens E , Overvad K , Panico S , Thompson S , Fournier A , Clavel‐Chapelon F , Canonico M , et al. EPIC‐Heart: the cardiovascular component of a prospective study of nutritional, lifestyle and biological factors in 520,000 middle‐aged participants from 10 European countries. Eur J Epidemiol. 2007;22:129–141. DOI: 10.1007/s10654-006-9096-8. 17295097

[27] Riboli E , Hunt KJ , Slimani N , Ferrari P , Norat T , Fahey M , Charrondière UR , Hémon B , Casagrande C , Vignat J , et al. European Prospective Investigation into Cancer and Nutrition (EPIC): study populations and data collection. Public Health Nutr. 2002;5:1113–1124. DOI: 10.1079/PHN2002394. 12639222

[28] Sharp SJ , Poulaliou M , Thompson SG , White IR , Wood AM . A review of published analyses of case‐cohort studies and recommendations for future reporting. PLoS One. 2014;9:e101176. DOI: 10.1371/journal.pone.0101176. 24972092 PMC4074158

[29] InterAct Consortium , Langenberg C , Sharp S , Forouhi NG , Franks PW , Schulze MB , Kerrison N , Ekelund U , Barroso I , et al. Design and cohort description of the InterAct Project: an examination of the interaction of genetic and lifestyle factors on the incidence of type 2 diabetes in the EPIC Study. Diabetologia. 2011;54:2272–2282. 10.1007/s00125-011-2182-9. 21717116 PMC4222062

[30] Kaaks R , Slimani N , Riboli E . Pilot phase studies on the accuracy of dietary intake measurements in the EPIC project: overall evaluation of results. European Prospective Investigation into Cancer and Nutrition. Int J Epidemiol. 1997;26(suppl 1):S26–S36. 9126531 10.1093/ije/26.suppl_1.s26

[31] Slimani N , Deharveng G , Unwin I , Southgate DAT , Vignat J , Skeie G , Salvini S , Parpinel M , Møller A , Ireland J , et al. The EPIC nutrient database project (ENDB): a first attempt to standardize nutrient databases across the 10 European countries participating in the EPIC study. Eur J Clin Nutr. 2007;61:1037–1056. DOI: 10.1038/sj.ejcn.1602679. 17375121

[32] van Bakel MME , Kaaks R , Feskens EJM , Rohrmann S , Welch AA , Pala V , Avloniti K , van der Schouw YT , van der A DL , Du H , et al. Dietary glycaemic index and glycaemic load in the European Prospective Investigation into Cancer and Nutrition. Eur J Clin Nutr. 2009;63(suppl 4):S188–S205. DOI: 10.1038/ejcn.2009.81. 19888274

[33] Willett WC , Howe GR , Kushi LH . Adjustment for total energy intake in epidemiologic studies. Am J Clin Nutr. 1997;65:1220S–1228S. DOI: 10.1093/ajcn/65.4.1220S. 9094926

[34] Linseisen J , Welch AA , Ocké M , Amiano P , Agnoli C , Ferrari P , Sonestedt E , Chajès V , Bueno‐de‐Mesquita HB , Kaaks R , et al. Dietary fat intake in the European Prospective Investigation into Cancer and Nutrition: results from the 24‐h dietary recalls. Eur J Clin Nutr. 2009;63(suppl 4):S61–S80. DOI: 10.1038/ejcn.2009.75. 19888281

[35] Bechthold A , Boeing H , Schwedhelm C , Hoffmann G , Knüppel S , Iqbal K , De Henauw S , Michels N , Devleesschauwer B , Schlesinger S , et al. Food groups and risk of coronary heart disease, stroke and heart failure: a systematic review and dose‐response meta‐analysis of prospective studies. Crit Rev Food Sci Nutr. 2019;59:1071–1090. DOI: 10.1080/10408398.2017.1392288. 29039970

[36] Soedamah‐Muthu SS , de Goede J . Dairy Consumption and cardiometabolic diseases: systematic review and updated meta‐analyses of prospective cohort studies. Curr Nutr Rep. 2018;7:171–182. DOI: 10.1007/s13668-018-0253-y. 30406514 PMC6244750

[37] Chowdhury R , Warnakula S , Kunutsor S , Crowe F , Ward HA , Johnson L , Franco OH , Butterworth AS , Forouhi NG , Thompson SG , et al. Association of dietary, circulating, and supplement fatty acids with coronary risk: a systematic review and meta‐analysis. Ann Intern Med. 2014;160:398–406. DOI: 10.7326/M13-1788. 24723079

[38] de Souza RJ , Mente A , Maroleanu A , Cozma AI , Ha V , Kishibe T , Uleryk E , Budylowski P , Schünemann H , Beyene J , et al. Intake of saturated and trans unsaturated fatty acids and risk of all cause mortality, cardiovascular disease, and type 2 diabetes: systematic review and meta‐analysis of observational studies. BMJ. 2015;351:h3978. DOI: 10.1136/bmj.h3978. 26268692 PMC4532752

[39] Sluijs I , Beulens JWJ , van der Schouw YT , van der A DL , Buckland G , Kuijsten A , Schulze MB , Amiano P , Ardanaz E , Balkau B , et al. Dietary glycemic index, glycemic load, and digestible carbohydrate intake are not associated with risk of type 2 diabetes in eight European countries. J Nutr. 2013;143:93–99. DOI: 10.3945/jn.112.165605. 23190759

[40] Davy B , Jahren H . New markers of dietary added sugar intake. Curr Opin Clin Nutr Metab Care. 2016;19:282–288. DOI: 10.1097/MCO.0000000000000287. 27137898 PMC4918090

[41] Key TJ , Appleby PN , Bradbury KE , Sweeting M , Wood A , Johansson I , Kühn T , Steur M , Weiderpass E , Wennberg M , et al. Consumption of meat, fish, dairy products, and eggs and risk of ischemic heart disease. Circulation. 2019;139:2835–2845. DOI: 10.1161/CIRCULATIONAHA.118.038813. 31006335 PMC6629175

[42] Brassard D , Tessier‐Grenier M , Allaire J , Rajendiran E , She Y , Ramprasath V , Gigleux I , Talbot D , Levy E , Tremblay A , et al. Comparison of the impact of SFAs from cheese and butter on cardiometabolic risk factors: a randomized controlled trial. Am J Clin Nutr. 2017;105:800–809. DOI: 10.3945/ajcn.116.150300. 28251937

[43] Hjerpsted J , Leedo E , Tholstrup T . Cheese intake in large amounts lowers LDL‐cholesterol concentrations compared with butter intake of equal fat content. Am J Clin Nutr. 2011;94:1479–1484. DOI: 10.3945/ajcn.111.022426. 22030228

[44] Guasch‐Ferre M , Satija A , Blondin SA , Janiszewski M , Emlen E , O'Connor LE , Campbell WW , Hu FB , Willett WC , Stampfer MJ . Meta‐analysis of randomized controlled trials of red meat consumption in comparison with various comparison diets on cardiovascular risk factors. Circulation. 2019;139:1828–1845. DOI: 10.1161/CIRCULATIONAHA.118.035225. 30958719

[45] Mozaffarian D , Wu JHY . Flavonoids, dairy foods, and cardiovascular and metabolic health: a review of emerging biologic pathways. Circ Res. 2018;122:369–384. DOI: 10.1161/CIRCRESAHA.117.309008. 29348256 PMC5781235

[46] Wolk A . Potential health hazards of eating red meat. J Intern Med. 2017;281:106–122. DOI: 10.1111/joim.12543. 27597529

[47] Micha R , Khatibzadeh S , Shi P , Fahimi S , Lim S , Andrews KG , Engell RE , Powles J , Ezzati M , Mozaffarian D , et al. Global, regional, and national consumption levels of dietary fats and oils in 1990 and 2010: a systematic analysis including 266 country‐specific nutrition surveys. BMJ. 2014;348:g2272. DOI: 10.1136/bmj.g2272. 24736206 PMC3987052

