# Supplementary material for: Dietary Fatty Acids, Macronutrient Substitutions, Food Sources and Incidence of Coronary Heart Disease: Findings From the EPIC‐CVD Case‐Cohort Study Across Nine European Countries
Source: J Am Heart Assoc. 2021 Nov 19;10(23):e019814. doi: 10.1161/JAHA.120.019814 (PMC9075396; doi:10.1161/JAHA.120.019814)
Supplement: Supplementary file 1 — Tables S1–S12 Figures S1–S5 [file JAH3-10-e019814-s001.pdf]

SUPPLEMENTAL MATERIAL

Dietary fatty acids, macronutrient substitutions, food sources and incidence of coronary heart disease: findings from the EPIC-CVD case-cohort study across nine European countries

Marinka Steur et al

Correspondence to: Nita G. Forouhi, FFPH, MRC Epidemiology Unit, University of Cambridge School of Clinical Medicine, Box 285, Institute of Metabolic Science, Cambridge Biomedical Campus, Cambridge, CB2 0QQ United Kingdom. E-mail: nita.forouhi@mrc-epid.cam.ac.uk

Table S1. Baseline biomedical risk factors in lowest (Q1) and highest (Q5) quintiles of dietary total fatty acids, saturated, monounsaturated and polyunsaturated fatty acids (%TEI) in the EPIC-CVD case-cohort study subcohort, among a subset of participants with available biomarkers, adjusted for age, sex and study centre.

			Total fatty acids		SFAs		MUFAs		PUFAs	
		Subcohort	Q1 (≤29.9)	Q5 (≥40.2)	Q1 (≤11.2)	Q5 (≥17.1)	Q1 (≤11.3)	Q5 (≥18.0)	Q1 (≤4.3)	Q5 (≥7.5)
**Lipids (mmol/l)***	**N**	16,013	3,219	3,197	3,261	3,137	3,208	3,282	3,246	3,218
	**TC**	5.94 (5.92, 5.95)	5.96 (5.94, 5.97)	5.91 (5.89, 5.93)	5.92 (5.90, 5.94)	5.94 (5.92, 5.96)	6.01 (5.99, 6.03)	5.88 (5.85, 5.90)	5.90 (5.88, 5.92)	5.95 (5.93, 5.97)
	**HDL-C**	1.49 (1.48, 1.49)	1.50 (1.50, 1.51)	1.47 (1.46, 1.47)	1.47 (1.47, 1.48)	1.50 (1.49, 1.51)	1.52 (1.51, 1.53)	1.45 (1.44, 1.46)	1.48 (1.47, 1.49)	1.50 (1.49, 1.50)
	**Non HDL-C**	4.45 (4.43, 4.47)	4.45 (4.44, 4.47)	4.44 (4.42, 4.47)	4.45 (4.43, 4.47)	4.44 (4.42, 4.46)	4.49 (4.47, 4.51)	4.42 (4.39, 4.45)	4.42 (4.40, 4.44)	4.46 (4.44, 4.48)
	**Triglycerides †**	1.15 (1.14, 1.16)	1.15 (1.14, 1.16)	1.12 (1.11, 1.14)	1.11 (1.09, 1.12)	1.19 (1.18, 1.20)	1.19 (1.18, 1.21)	1.06 (1.05, 1.07)	1.10 (1.09, 1.11)	1.15 (1.14, 1.16)
	**TC:HDL-C ratio†**	4.08 (4.06, 4.10)	4.05 (4.03, 4.07)	4.12 (4.10, 4.15)	4.10 (4.07, 4.12)	4.06 (4.03, 4.08)	4.04 (4.02, 4.07)	4.12 (4.09, 4.16)	4.07 (4.04, 4.09)	4.06 (4.04, 4.09)
**Blood Pressure (mm Hg)***	**N**	12,696	2,597	2,490	1,905	2,826	2,897	2,002	2,484	2,395
	**SBP**	132.1 (131.8, 132.4)	132.2 (131.9, 132.6)	131 (130.5, 131.4)	131.4 (131, 131.8)	132.9 (132.5, 133.2)	132.4 (132.0, 132.7)	128.8 (128.3, 129.4)	131.3 (131, 131.7)	131.4 (131.1, 131.8)
	**DBP**	81.5 (81.3, 81.7)	81.1 (80.9, 81.3)	81.3 (81.1, 81.6)	81.2 (80.9, 81.4)	81.8 (81.6, 82.0)	80.8 (80.6, 81.0)	80.7 (80.3, 81.0)	81.4 (81.2, 81.6)	81.1 (80.9, 81.3)
**CRP (mg/l) †**	**N**	16,016	3,218	3,201	3,267	3,140	3,206	3,283	3,244	3,244
		1.16 (1.14, 1.17)	1.17 (1.15, 1.19)	1.14 (1.12, 1.17)	1.20 (1.18, 1.23)	1.11 (1.09, 1.13)	1.17 (1.14, 1.19)	1.18 (1.15, 1.21)	1.15 (1.13, 1.17)	1.15 (1.13, 1.17)
**HbA1c (%)***	**N**	16,426	3,264	3,303	3,310	3,275	3,256	3,316	3,273	3,308
		5.52 (5.51, 5.53)	5.52 (5.51, 5.53)	5.56 (5.54, 5.57)	5.52 (5.51, 5.53)	5.53 (5.52, 5.54)	5.52 (5.51, 5.53)	5.54 (5.53, 5.56)	5.53 (5.52, 5.54)	5.50 (5.49, 5.52)

CRP, C-reactive protein; DBP, diastolic blood pressure; IQR, interquartile range; HDL-C, high-density lipoprotein cholesterol; MUFA, monounsaturated fatty acids; PUFA, polyunsaturated fatty acid; SBP, systolic blood pressure; SFA, saturated fatty acid; TC, total cholesterol; CI, confidence interval; %TEI, percentage of total energy intake.
Systolic and diastolic blood pressure measurements: In most centres, systolic and diastolic blood pressure was measured in duplicate in sitting position.[26] Where available, we used the average of both measurements.

Biochemical measurements: Non-fasted blood samples obtained at baseline were stored at the International Agency for Research on Cancer (IARC) or local biobanks.[29] Serum concentrations of total cholesterol (TC), HDL-Cholesterol (HDL-C), triglycerides and high-sensitivity C-reactive protein (CRP), and erythrocyte haemoglobin A1c

(HbA1c) were measured by Stichting Huisartsen Laboratorium (Etten-Leur; the Netherlands), using Cobas enzymatic assays (Roche Diagnostics, Mannheim, Germany) on a Roche/Hitachi Modular P Analyzer for all biomarkers except HbA1c, which was measured with a Tosoh-G8 HPLC (Tosoh Bioscience, Japan). [29]
Means (95% CIs) were estimated from multivariable linear regression, adjusted for age, sex and study centre, among participants in the overall subcohort with measured data on all presented lipids (n=16,013), SBP and DBP (n=12,696), CRP (n=16,016) and HbA1c (n=16,426).
* All values are means (95% CI), unless specified otherwise.
† Geometric means (95% CI).

Table S2. HRs (95% CIs) of CHD across quintiles of dietary total fat, saturated, monounsaturated and polyunsaturated fatty acid intake (%TEI) and tests for trend across categories: EPIC-CVD case-cohort study.

		Q1 (lowest intake)	Q2	Q3	Q4	Q5 (highest intake)	P for trend
**Total fat**	**Median (subcohort)**	27.4	31.8	35.0	38.3	44.0	
	**HR (95% CI)**	1.00 (ref)	0.97 (0.87, 1.08)	0.95 (0.86, 1.06)	0.92 (0.81, 1.05)	1.02 (0.89, 1.16)	0.859
**SFA**	**Median (subcohort)**	9.7	12.2	14.0	15.9	18.7	
	**HR (95% CI)**	1.00 (ref)	0.88 (0.79, 0.98)	0.87 (0.77, 0.98)	0.87 (0.74, 1.02)	0.86 (0.75, 0.99)	0.173
**MUFA†**		10.1	12.2	14.0	16.2	21.1	
	**HR (95% CI)**	1.00 (ref)	0.96 (0.86, 1.09)	0.99 (0.86, 1.13)	1.06 (0.85, 1.33)	0.98 (0.82, 1.19)	0.466
**PUFA**		3.9	4.7	5.6	6.7	9.0	
	**HR (95% CI)**	1.00 (ref)	0.98 (0.88, 1.09)	1.00 (0.86, 1.15)	0.99 (0.87, 1.13)	0.98 (0.87, 1.10)	0.920

CI, confidence interval; HR, hazard ratio; SFA, saturated fatty acid; MUFA, monounsaturated fatty acid; PUFA, polyunsaturated fatty acid.
Hazard ratios (HRs) and 95% confidence intervals (CIs) across quintiles of dietary SFAs, MUFAs and PUFAs, where quintiles were based on the overall subcohort, were analysed within each country separately, with age as the underlying time variable and the baseline hazard stratified by sex. Country-specific HRs (95% CIs) were combined in multivariate random-effects meta-analysis to obtain pooled effect estimates and 95% CIs. The multivariable-adjusted HR included adjustment for age at recruitment (years), centre, energy intake (kcal/day), education (low, medium, high), smoking (never, former, current), physical activity (inactive, moderately inactive, moderately active, active), alcohol intake (0, 0-6, 6-12, 12-13, >24 g/day), dietary fibre (g/day, continuous), fruit and vegetable consumption (g/day, continuous), body-mass index (kg/m^2, continuous), reported history of diabetes, hypertension and hyperlipidemia. P for trend was assessed by assigning participants the median dietary intake level in their respective quintile (based on the overall subcohort) and analysing as a continuous exposure.
† Greece was excluded in analysis of MUFA with CHD, because there were no CHD cases in the reference category (Q1)

Table S3. Contribution of food groups (%) to dietary saturated fatty acid intake (%TEI) in the EPIC-CVD case-cohort study subcohort.

		Subcohort (n=16,730)	Greece (n=1,201)	Spain (n=3,639)	Italy (n=1,992)	France (n=551)	UK (n=1,076)	Netherlands (n=1,356)	Germany (n=1,995)	Denmark (n=2,005)	Sweden (n=2,915)
Total SFAs from all foods (%TEI)		14.0 (11.7-16.4)	13.2 (11.6-14.9)	11.6 (9.6-13.8)	12.4 (10.7-14.2)	16.1 (13.7-18.4)	13.6 (11.7-15.7)	14.9 (13.1-16.7)	15.6 (13.7-17.7)	15.0 (12.9-16.9)	16.0 (14.0-18.3)
Dairy products		32.0 (22.6-42.3)	41.4 (31.8-50.2)	29.7 (16.6-43.2)	38.2 (29.4-47.4)	37.7 (27.8-46.4)	27.8 (20.3-36.6)	34.5 (26.6-43.9)	28.9 (21.1-37.4)	28.7 (20.1-37.8)	31.2 (23.6-39.3)
	Milk	4.8 (1.0-11.3)	4.6 (1.0-10.1)	9.1 (1.2-18.5)	3.8 (0.0-8.2)	0.3 (0.0-3.6)	10.5 (3.2-17.4)	6.6 (2.6-11.4)	1.4 (0.1-5.7)	4.2 (1.2-9.7)	4.7 (2.1-9.5)
	Yoghurt / thick fermented milk	0.9 (0.0-3.3)	1.7 (0.7-3.7)	0.0 (0.0-1.7)	0.3 (0.0-2.1)	1.3 (0.3-3.0)	0.3 (0.0-1.1)	0.6 (0.2-1.5)	2.1 (0.6-4.9)	1.6 (0.3-6.7)	1.9 (0.0-5.9)
	Cheese	17.0 (8.5-28.1)	30.5 (21.1-39.7)	10.6 (0.6-26.6)	30.4 (22.0-40.1)	25.8 (15.5-35.6)	10.8 (5.5-16.3)	19.2 (12.1-27.5)	13.5 (8.2-20.3)	14.7 (8.5-23.0)	15.2 (8.8-22.9)
Added fats		20.5 (13.5-29.4)	28.4 (22.9-35.1)	15.5 (10.4-22.6)	20.6 (15.9-26.4)	12.8 (7.3-19.6)	16.8 (10.5-25.1)	21.0 (14.9-29.3)	20.3 (13.5-28.8)	22.8 (13.0-31.7)	25.6 (17.0-36.3)
	vegetable oils*	3.3 (0.7-14.0)	25.2 (20.1-31.9)	13.4 (9.0-19.6)	16.3 (12.2-21.6)	2.2 (1.4-3.5)	2.7 (1.4-4.5)	1.2 (0.2-2.6)	1.7 (1.0-3.0)	0.7 (0.3-2.2)	0.0 (0.0-0.7)
	butter	0.1 (0.0-3.8)	0.1 (0.0-1.2)	0.0 (0.0-0.0)	1.0 (0.0.2-3)	5.9 (0.6-14.2)	0.6 (0-9.5.0)	4.7 (2.8-8.9)	8.2 (1.6-20.2)	0.0 (0.0-0.0)	0.0 (0.0-0.3)
	margarines	3.0 (0.1-13.5)	0.9 (0.1-3.4)	0.0 (0.0-0.6)	0.1 (0.0-0.2)	0.0 (0.0-2.3)	6.6 (2.0-12.5)	7.7 (3.7-13.0)	3.6 (0.8-8.8)	15.0 (5.5-26.2)	21.1 (13.2-31.3)
Meat		15.7 (10.1-23.2)	10.6 (7.8-14.3)	19.5 (12.4-29.2)	11.0 (7.6-15.3)	13.5 (9.4-18.8)	10.5 (6.3-15.5)	15.5 (10.9-21.4)	17.6 (11.1-24.5)	24.3 (18.6-31.0)	15.3 (10.7-20.8)
	red meat	5.2 (2.7-9.7)	7.3 (4.9-10.3)	4.4 (2.1-7.7)	4.2 (2.5-6.3)	6.0 (2.7-10.4)	4.2 (1.9-7.2)	8.3 (5.2-12.2)	2.8 (1.7-4.5)	15.8 (11.7-20.7)	4.4 (2.3-7.6)
	processed	6.1 (2.6-11.2)	0.3 (0.0-0.7)	8.5 (3.7-15.6)	3.8 (2.0-6.7)	4.8 (2.4-7.6)	4.5 (2.3-7.4)	5.1 (2.7-8.6)	12.9 (7.4-19.2)	5.9 (3.4-9.3)	7.6 (4.2-11.6)
	poultry	1.1 (0.4-2.3)	1.7 (1.0-3.0)	2.7 (1.4-5.0)	1.2 (0.6-2.2)	1.1 (0.2-2.1)	0.5 (0.2-1.2)	0.6 (0.3-1.2)	0.7 (0.4-1.4)	0.9 (0.5-1.6)	0.5 (0.0-1.4)
Cakes and biscuits		6.5 (2.7-12.7)	2.5 (1.1-4.4)	7.3 (0.0-20.0)	8.1 (3.7-14.3)	7.0 (3.2-11.7)	10.0 (5.7-16.9)	5.6 (3.1-8.8)	10.3 (5.4-16.6)	4.1 (2.0-7.8)	6.9 (3.8-11.3)
Sugar and confectionary		2.8 (0.7-6.1)	2.5 (0.6-5.0)	0.0 (0.0-1.1)	4.0 (1.8-7.5)	1.5 (0.2-7.0)	7.1 (3.5-12.9)	5.1 (2.9-8.1)	2.3 (1.0-5.0)	3.2 (1.8-6.0)	3.5 (1.7-6.6)
Cereal and cereal products		2.9 (1.9-4.5)	2.1 (1.5-2.9)	2.7 (1.7-4.1)	5.2 (3.2-7.7)	3.3 (2.2-4.6)	4.5 (2.7-7.4)	4.0 (2.9-5.3)	2.4 (1.6-3.7)	2.8 (2.0-3.8)	2.5 (1.8-3.5)
Egg and egg products		1.6 (0.8-2.8)	1.3 (0.8-2.0)	2.7 (1.5-4.5)	1.8 (1.1-2.7)	1.9 (1.1-3.1)	1.0 (0.5-2.1)	1.2 (0.7-2.0)	1.4 (0.7-2.2)	1.8 (1.1-3.1)	0.6 (0.2-1.8)
Condiments and sauces		1.1 (0.4-2.8)	1.9 (0.7-3.7)	0.5 (0.2-1.0)	0.6 (0.2-1.2)	4.9 (3.1-7.0)	2.6 (1.3-4.7)	1.0 (0.5-1.7)	1.9 (1.0-3.5)	1.6 (0.7-3.2)	1.8 (0.4-4.8)
Fish and shellfish		1.0 (0.4-2.0)	0.3 (0.2-0.6)	1.9 (0.9-3.5)	0.8 (0.4-1.4)	0.7 (0.4-1.2)	0.8 (0.3-1.4)	0.4 (0.1-0.8)	0.9 (0.4-1.6)	1.9 (1.1-3.1)	0.9 (0.3-2.0)
Nuts and seeds		0.1 (0.0-0.7)	0.8 (0.1-1.7)	0.0 (0.0-0.6)	0.0 (0.0-0.1)	0.6 (0.0-1.4)	0.4 (0.0-1.1)	1.3 (0.4-3.0)	0.2 (0.1-0.9)	0.2 (0.1-0.3)	0.0 (0.0-0.1)

SFAs, saturated fatty acids; %TEI, percentage of total energy intake.

All values are median (interquartile range), in %TEI (for total SFAs) or in % contribution to dietary SFA intakes (for foods).
* Includes all vegetable oils evaluated by country -specific dietary questionnaires, including olive oil where applicable.

Table S4. Pearson partial correlations (95% CI) in the EPIC-CVD case-cohort study subcohort (n=16,730).

	SFA	MUFA	PUFA	Carbohydrates	Animal protein	Plant protein	Total energy
**SFA**	1						
**MUFA**	0.54 (0.36, 0.68)	1					
**PUFA**	0.03 (-0.06, 0.11)	0.26 (-0.01, 0.51)	1				
**Carbohydrates**	-0.70 (-0.76, -0.63)	-0.79 (-0.85, -0.70)	-0.40 (-0.48, -0.32)	1			
**Animal protein**	0.23 (0.07, 0.37)	0.15 (0.05, 0.24)	0.03 (-0.07, 0.12)	-0.58 (-0.65, -0.50)	1		
**Plant protein**	-0.56 (-0.62, -0.48)	-0.32 (-0.42, -0.23)	0.00 (-0.08, 0.09)	0.42 (0.33, 0.50)	-0.34 (-0.43, -0.24)	1	
**Total energy**	0.20 (0.15, 0.25)	0.09 (0.01, 0.16)	0.05 (-0.01, 0.10)	-0.03 (-0.09, 0.03)	-0.16 (-0.22, -0.11)	-0.22 (-0.31, -0.13)	1

CI, confidence interval; MUFA, monounsaturated fatty acid; PUFA, polyunsaturated fatty acid; SFA, saturated fatty acid.
All macronutrients are expressed in % of total energy intake. Pearson partial correlations, adjusted for age and sex, were calculated in the subcohort of each country. Fisher's Z transformation was then used to transform country-specific correlation coefficients r (95% CIs) to obtain a normally distributed variable, i.e. using z = 0.5*ln((1+r)/1-r)). Z-transformed correlation coefficients r (95% CIs) were subsequently pooled using random effects meta-analysis, and back-transformation was applied to obtain an overall correlation coefficient r (95% CI).

Table S5. HRs (95% CIs) of CHD and dietary total fatty acids, saturated, monounsaturated and polyunsaturated fatty acids (per 5 %TEI) with progressive adjustment for confounders in the EPIC-CVD case-cohort study.

	Model 1			Model 2			Model 3			Model 4		
	HR (95% CI)	P	I^2	HR (95% CI)	P	I^2	HR (95% CI)	P	I^2	HR (95% CI)	P	I^2
**Total fatty acids**	1.04 (0.99, 1.09)	0.083	63	1.01 (0.97, 1.06)	0.554	51	1.00 (0.97, 1.04)	0.779	18	1.00 (0.96, 1.04)	0.992	25
**SFA**	1.06 (0.97, 1.16)	0.205	68	1.00 (0.93, 1.08)	0.907	53	0.97 (0.90, 1.05)	0.411	42	0.99 (0.91, 1.08)	0.815	45
**MUFA**	1.10 (0.98, 1.22)	0.107	75	1.04 (0.95, 1.13)	0.382	57	1.03 (0.96, 1.10)	0.449	29	1.01 (0.94, 1.08)	0.806	27
**PUFA**	1.03 (0.93, 1.14)	0.567	36	1.03 (0.94, 1.12)	0.512	20	1.07 (0.99, 1.16)	0.074	2	1.03 (0.95, 1.12)	0.471	0

CI, confidence interval; HR, hazard ratio; SD, standard deviation; SFA, saturated fatty acid; MUFA, monounsaturated fatty acid; PUFA, polyunsaturated fatty acid.
Hazard ratios (HRs) and 95% confidence intervals (CIs) for each 5% higher contribution to total energy intake from dietary SFAs, MUFAs and PUFAs were analysed within each country separately, with age as the underlying time variable and the baseline hazard stratified by sex. Country-specific HRs (95% CIs) were combined in multivariate random-effects meta-analysis to obtain pooled effect estimates and 95% CIs. The multivariable-adjusted HR included adjustment for (model 1:) age at recruitment (years), centre, energy intake (kcal/day), (model 2:) education (low, medium, high), smoking (never, former, current), physical activity (inactive, moderately inactive, moderately active, active), alcohol intake (0, 0-6, 6-12, 12-13, >24 g/day), (model 3:) dietary fibre (g/day, continuous), fruit and vegetable consumption (g/day, continuous), (model 4:)), body-mass index (kg/m^2, continuous), reported history of diabetes, hypertension and hyperlipidemia.

Table S6. Associations with coronary heart disease of substituting 5% total energy intake from carbohydrates for 5% total energy from saturated fatty acids by thirds of energy-adjusted glycaemic index in the EPIC-CVD case-cohort study.

GI category (range energy-adjusted GI)	N cases/ N total	HR (95% CI)*	P	P interaction †
Low (34.2-54.4)	2,642 / 7,933	0.93 (0.79, 1.11)	0.430	0.579
Medium (54.4-57.5)	3,561 / 8,815	1.01 (0.80, 1.29)	0.904	
High (57.5-77.8)	4,289 / 9,353	1.21 (1.02, 1.43)	0.030	

HR, hazard ratio; CI, confidence interval; GI, glycaemic index;
Hazard ratios (HRs) and 95% confidence intervals (CIs) for each 5% higher contribution to total energy intake from dietary carbohydrates to substitute for 5% lower energy intake from SFAs among participants with low, medium and high glycaemic index were analysed within each country separately, with age as the underlying time variable and the baseline hazard stratified by sex. The multivariable-adjusted HR included adjustment for all macronutrients except SFA, i.e. MUFAs, PUFAs, carbohydrates, plant protein, animal protein and mixed-origin protein (all in %TEI), and centre, energy intake (kcal/day), education (low, medium, high), smoking (never, former, current), physical activity (inactive, moderately inactive, moderately active, active), alcohol intake (0, 0-6, 6-12, 12-13, >24 g/day), dietary fibre (g/day, continuous), fruit and vegetable consumption (g/day, continuous), body-mass index (kg/m^2, continuous), pre-existing diabetes, hypertension and hyperlipidemia.. Country-specific HRs (95% CIs) were combined in multivariate random-effects meta-analysis to obtain pooled effect estimates and 95% CIs. Low, medium and high GI were defined by thirds of the distribution in the overall subcohort. Not adjusting associations across strata of GI for fibre intake in strata-specific analyses did not affect the results.
* France was excluded from these analysis to aid model convergence. Age at recruitment was not included as covariate in these models to aid model convergence in country-specific analysis within GI strata – analysis including age at recruitment as covariate yielded similar results where models did converge.
† P-values for interaction: reported P-value is from the model including the interaction term between GI and carbohydrate intake and not adjusted for dietary fibre consumption, and meta-analysis of the interaction term only.

Table S7. Stratified analysis of dietary saturated, monounsaturated and polyunsaturated fatty acids (per 5%TEI) and CHD in the EPIC-CVD case-cohort study.

		SFAs			MUFAs			PUFAs		
**Effect modifier**		**HR (95% CI)**	**P**	**P interaction**	**HR (95% CI)**	**P**	**P interaction**	**HR (95% CI)**	**P**	**P interaction**
**Sex***				NA/0.425			NA/0.075			NA/0.117
	**Men**	1.02 (0.94, 1.10)	0.708		1.04 (0.95, 1.14)	0.353		1.00 (0.90, 1.11)	0.980	
	**Women (all)**	0.92 (0.80, 1.06)	0.255		0.96 (0.87, 1.06)	0.395		1.08 (0.92, 1.26)	0.366	
	**Women (mixed-sex centres)**	0.95 (0.81, 1.12)	0.538	0.456	0.97 (0.87, 1.07)	0.514	0.100	1.15 (0.99, 1.34)	0.064	0.106
**Age***				0.091/0.367 †			0.749/0.4967†			0.480/0.161 †
	**<52.4**	0.89 (0.75, 1.05)	0.155		0.98 (0.85, 1.13)	0.751		0.90 (0.70, 1.17)	0.442	
	**≥52.4**	1.01 (0.92, 1.11)	0.825		1.03 (0.95, 1.11)	0.432		1.04 (0.94, 1.15)	0.499	
**Plausibility of self-reported energy intake***				NA/0.956			NA/0.557			NA/0.460
	**Under**	1.01 (0.89, 1.15)	0.845		1.06 (0.94, 1.19)	0.343		0.94 (0.80, 1.10)	0.446	
	**Plausible**	0.98 (0.85, 1.12)	0.731		1.03 (0.90, 1.18)	0.652		1.04 (0.92, 1.17)	0.536	
	**Over**	0.93 (0.67, 1.30)	0.683		1.07 (0.82, 1.40)	0.610		1.44 (0.91, 2.30)	0.122	
**Pre-existing diabetes/ hypertension/ hyperlipidemia**				NA/0.956			NA/0.975			NA/0.090
	**No/unknown**	0.99 (0.88, 1.13)	0.928		1.00 (0.92, 1.08)	0.965		0.99 (0.89, 1.10)	0.847	
	**yes**	1.00 (0.87, 1.15)	0.996		1.00 (0.91, 1.08)	0.918		1.11 (0.98, 1.26)	0.092	

CI, confidence interval; HR, hazard ratio; SFA, saturated fatty acid; %TEI, percentage contribution to total energy intake.
Hazard ratios (HRs) and 95% confidence intervals (CIs) for each 5% higher contribution to total energy intake from dietary SFAs, MUFAs and PUFAs by categories of potential effect modifiers were analysed, with age as the underlying time variable and the baseline hazard stratified by sex. The multivariable-adjusted HR included adjustment for age at recruitment, centre, energy intake (kcal/day), education (low, medium, high), smoking (never, former, current), physical activity (inactive, moderately inactive, moderately active, active), alcohol intake (0, 0-6, 6-12, 12-13, >24 g/day), dietary fibre (g/day, continuous), fruit and vegetable consumption (g/day, continuous), body-mass index (kg/m^2, continuous), reported history of diabetes, hypertension and hyperlipidemia.
* France was excluded from analysis of sex, age and energy reporting because <10 cases in at least one reference category.
† P-value for interaction, investigated with effect modifier modelled as continuous / categorical covariate in the models.

Table S8. Stratified analysis of associations with CHD of substituting 5% energy from monounsaturated fatty acids, polyunsaturated fatty acids, and carbohydrates for energy from SFAs in the EPIC-CVD case-cohort study.

		MUFAs			PUFAs			Carbohydrates		
**Effect modifier**		**HR (95% CI)**	**P**	**P interaction**	**HR (95% CI)**	**P**	**P interaction**	**HR (95% CI)**	**P**	**P interaction**
**Sex***				0.0866			0.1319			0.9133
	**Men**	1.15 (0.94, 1.39)	0.173		0.90 (0.75, 1.08)	0.264		1.01 (0.90, 1.13)	0.918	
	**Women (all)**	1.11 (0.89, 1.39)	0.332		1.14 (0.93, 1.41)	0.214		1.08 (0.92, 1.28)	0.346	
	**Women (mixed-sex centres)**	1.06 (0.84, 1.36)	0.611	0.1161	1.15 (0.92, 1.44)	0.209	0.1222	1.05 (0.87, 1.27)	0.621	0.9308
**Age***				0.8290/0.7333†			0.3383/0.1165†			0.2130/0.1759†
	**<52.4**	1.63 (1.19, 2.25)	**0.003**		0.93 (0.64, 1.35)	0.701		1.27 (1.07, 1.50)	**0.005**	
	**≥52.4**	1.09 (0.92, 1.29)	0.316		0.97 (0.83, 1.14)	0.740		1.00 (0.91, 1.10)	0.966	
**Plausibility of self-reported energy intake***				0.6239			0.5984			0.8945
	**Under reporting**	1.12 (0.82, 1.52)	0.475		0.95 (0.71, 1.27)	0.731		1.03 (0.71, 1.27)	0.790	
	**Plausible**	1.24 (0.98, 1.57)	0.847		1.02 (0.82, 1.27)	0.847		1.08 (0.94, 1.25)	0.280	
	**Over reporting**	1.08 (0.64, 1.80)	0.779		1.55 (0.77, 3.13)	0.218		1.07 (0.74, 1.55)	0.710	
**Pre-existing diabetes/ hypertension/ hyperlipidemia**				0.9289			0.0594			0.1903
	**No/unknown**	1.00 (0.77, 1.30)	0.998		0.93 (0.76, 1.30)	0.426		0.99 (0.83, 1.17)	0.871	
	**yes**	1.11 (0.87, 1.43)	0.408		1.07 (0.80, 1.42)	0.656		1.01 (0.83, 1.21)	0.948	

CI, confidence interval; HR, hazard ratio; MUFA, monounsaturated fatty acid; PUFA, polyunsaturated fatty acid; SFA, saturated fatty acid; %TEI, percentage contribution to total energy intake.
Hazard ratios (HRs) and 95% confidence intervals (CIs) for each 5% higher contribution to total energy intake from dietary MUFAs, PUFAs and carbohydrates to substitute for 5% lower energy intake from dietary SFAs by categories of potential effect modifiers were analysed, with age as the underlying time variable and the baseline hazard stratified by sex. The multivariable-adjusted HR included adjustment for all macronutrients except SFA, i.e. MUFAs, PUFAs, carbohydrates, plant protein, animal protein and mixed-origin protein (all in %TEI), and age at recruitment, centre, energy intake (kcal/day), education (low, medium, high), smoking (never, former, current), physical activity (inactive, moderately inactive, moderately active, active), alcohol intake (0, 0-6, 6-12, 12-13, >24 g/day), dietary fibre (g/day, continuous), fruit and vegetable consumption (g/day, continuous), body-mass index (kg/m^2, continuous), reported history of diabetes, hypertension and hyperlipidemia.
* France was excluded from analysis of sex, age and energy reporting because <10 cases in at least one reference category.
† P-value for interaction, investigated with effect modifier modelled as continuous / categorical covariate in the models.

Table S9 Associations with CHD of substituting dietary monounsaturated fatty acids, polyunsaturated fatty acids, carbohydrates, and saturated fatty acids from other foods, for saturated fatty acids from fermented dairy products, red meat, or butter (per 5%TEI) in the EPIC-CVD case-cohort study.

Food-specific SFAs to be substituted for (↓5%TEI)	Macronutrient substituting for SFAs from food (↑5%TEI)	HR (95% CI)	P	I^2
SFAs from fermented dairy (yoghurt + cheese)	MUFAs	1.18 (1.00, 1.40)	0.055	26
	PUFAs	1.07 (0.90, 1.27)	0.446	42
	Carbohydrates	1.13 (1.02, 1.25)	0.023	17
	SFAs from other foods (not fermented dairy)	1.20 (1.02, 1.40)	0.028	55
SFAs from red meat	MUFAs	0.74 (0.52, 1.07)	0.108	24
	PUFAs	0.67 (0.47, 0.95)	0.026	36
	Carbohydrates	0.70 (0.51, 0.97)	0.032	29
	SFAs from other foods (not red meat)	0.70 (0.52, 0.93)	0.014	24
SFAs from butter	MUFAs	1.01 (0.82, 1.25)	0.896	26
	PUFAs	0.89 (0.76, 1.04)	0.158	25
	Carbohydrates	0.92 (0.81, 1.05)	0.222	19
	SFAs from other foods (not butter)	0.86 (0.76, 0.98)	0.019	18

Hazard ratios (HRs) and 95% confidence intervals (CIs) for each 5% higher energy intake from MUFAs, PUFAs and carbohydrates to substitute for 5% lower energy intake from saturated fatty acids (SFAs) from specific foods were analysed within each country separately, with age as the underlying time variable and the baseline hazard stratified by sex. The multivariable-adjusted HR included adjustment for age at recruitment (years), centre, energy intake (kcal/day), education (low, medium, high), smoking (never, former, current), physical activity (inactive, moderately inactive, moderately active, active), alcohol intake (0, 0-6, 6-12, 12-13, >24 g/day), dietary fibre (g/day, continuous), fruit and vegetable consumption (g/day, continuous), body-mass index (kg/m^2, continuous), reported history of diabetes, hypertension and hyperlipidemia, and for total MUFAs, PUFAs, carbohydrates, plant-derived, animal-derived and mixed-origin protein, and the sum of SFAs from all other foods, all per 5%TEI. Country-specific HRs (95% CIs) were combined in univariate and multivariate random-effects meta-analysis to obtain pooled effect estimates and 95% CIs. The analysis included all 16,730 subcohort members and 10,529 CHD cases.

Table S10. Associations of dietary fatty acids (total and classes) with incident CHD by region in the EPIC-CVD case-cohort study.

	Overall			South	Central	North	
	HR (95% CI)	P	I^2 (95% CI)	HR (95% CI)	HR (95% CI)	HR (95% CI)	P for heterogeneity between regions
Intake of:							
Total fat[a]	1.00 (0.96, 2.04)	0.992	25 (0-65)	0.96 (0.91, 1.02)	1.03 (0.97, 1.09)	1.01 (0.96, 1.06)	0.192
SFAs[a]	0.99 (0.91, 1.08)	0.815	45 (0-75)	0.91 (0.79, 1.05)	1.06 (0.95, 1.18)	0.99 (0.93, 1.06)	**0.029**
MUFAs[a]	1.01 (0.94, 1.08)	0.806	27 (0-66)	0.96 (0.89, 1.04)	1.10 (0.95, 1.27)	1.06 (0.93, 1.19)	0.135
PUFAs[a]	1.03 (0.95, 1.12)	0.471	0 (0-65)	1.08 (0.97, 1.21)	0.95 (0.82, 1.11)	1.00 (0.84, 1.20)	0.400
Dietary SFAs to be substituted by:							
MUFAs[b]	1.14 (0.95, 1.36)	0.174	37 (0-71)	1.08 (0.88, 1.31)	1.03 (0.74, 1.45)	1.21 (0.88, 1.68)	0.808
PUFAs[b]	0.97 (0.82, 1.14)	0.680	51 (0-77)	1.17 (0.93, 1.47)	0.84 (0.69, 1.02)	1.00 (0.80, 1.25)	**0.039**
Carbohydrates[b]	1.04 (0.93, 1.15)	0.509	36 (0-71)	1.06 (0.91, 1.23)	0.94 (0.81, 1.10)	1.07 (0.94, 1.23)	0.441

CI, confidence interval; HR, hazard ratio; MUFA, monounsaturated fatty acid; PUFA, polyunsaturated fatty acid; SFA, saturated fatty acid.
South: Greece, Spain, Italy, France; Central: the Netherlands, United Kingdom, Germany; North: Denmark, Sweden.
[a]HR per 5% higher energy intake from exposure to substitute for energy from any other macronutrient source.
[b]HR (95% CI) per 5% higher energy intake from exposure to substitute 5% energy intake from SFA.
Hazard ratios (HRs) and 95% confidence intervals (CIs) were analysed within each country separately, with age as the underlying time variable and the baseline hazard stratified by sex. Country-specific HRs (95% CIs) were combined in multivariate random-effects meta-analysis to obtain pooled effect estimates and 95% CIs. The multivariable-adjusted HR included adjustment age at recruitment (years), centre, energy intake (kcal/day), education (low, medium, high), smoking (never, former, current), physical activity (inactive, moderately inactive, moderately active, active), alcohol intake (0, 0-6, 6-12, 12-13, >24 g/day), dietary fibre (g/day, continuous), fruit and vegetable consumption (g/day, continuous), body-mass index (kg/m^2, continuous), reported history of diabetes, hypertension and hyperlipidemia. In macronutrient-specific substitution analysis, models were additionally adjusted for all macronutrients except SFA, i.e. MUFAs, PUFAs, carbohydrates, plant protein, animal protein and mixed-origin protein (all in %TEI). P for heterogeneity between regions was obtained from fixed-effect meta-analysis by region. Region-specific HRs (95% CIs) were estimated by multivariate random-effects meta-analysis of country-specific effect estimates within each region.

Table S11. Associations of dietary fatty acids with CHD in the EPIC-CVD case-cohort study: sensitivity analyses

		Total fatty acids		SFAs		MUFAs		PUFAs	
	**N cases / total**	**HR (95% CI)***	**I^2 (95% CI)**	**HR (95% CI)***	**I^2 (95% CI)**	**HR (95% CI)***	**I^2 (95% CI)**	**HR (95% CI)***	**I^2 (95% CI)**
**Fatal CHD†**	1,614 / 17,688	1.01 (0.95, 1.08	0 (0-68)	1.00 (0.85, 1.16)	37 (0-72)	1.07 (0.94, 1.22)	0 (0-68)	1.00 (0.77, 1.29)	53 (0-79)
**Non-fatal CHD†**	8,878 / 24,592	1.00 (0.96, 1.04)	37 (0-72)	0.98 (0.90, 1.07)	46 (0-76)	1.00 (0.93, 1.08)	31 (0-69)	1.04 (0.95, 1.12)	0 (0-68)
**Excluding the first 2 years of follow-up**	9,479 / 25,637	0.99 (0.96, 1.03)	18 (0-60)	0.98 (0.89, 1.07)	48 (0-76)	0.99 (0.93, 1.06)	11 (0-52)	1.03 (0.95, 1.12)	1 (0-65)
**Excluding extreme energy intake reporters‡**	10,387 / 26,339	1.00 (0.96, 1.04)	38 (0-71)	0.99 (0.91, 1.08)	49 (0-76)	1.02 (0.93, 1.10)	42 (0-73)	1.02 (0.94, 1.11)	0 (0-65)

HR, hazard ratio; CHD, coronary heart disease; CI, confidence interval; SFA, saturated fatty acid; MUFA, monounsaturated fatty acid; PUFA, polyunsaturated fatty acid.

* HR (95% CI) per 5% higher energy intake from exposure to replace energy from any other macronutrient source.

† France was excluded from analyses of fatal and non-fatal CHD, because there were <10 incident fatal CHD cases. The incidence rates in the subcohort were 23.77 first non-fatal CHD events per 10,000 person-years, and 5.255 fatal CHD events per 10,000 person-years.

‡ Extreme energy intake was defined as total energy intake <500 or >3500 kcal/day for women, and <800 or >4000 kcal/day for men.

Hazard ratios (HRs) and 95% confidence intervals (CIs) were analysed within each country separately, with age as the underlying time variable and the baseline hazard stratified by sex. Country-specific HRs (95% CIs) were combined in multivariate random-effects meta-analysis to obtain pooled effect estimates and 95% CIs. The multivariable-adjusted HR included adjustment age at recruitment (years), centre, energy intake (kcal/day), education (low, medium, high), smoking (never, former, current), physical activity (inactive, moderately inactive, moderately active, active), alcohol intake (0, 0-6, 6-12, 12-13, >24 g/day), dietary fibre (continuous), fruit and vegetable consumption (continuous), body-mass index (continuous), reported history of diabetes, hypertension and hyperlipidemia.

Table S12. Associations of 5%TEI from monounsaturated fatty acids, polyunsaturated fatty acids, and carbohydrates to substitute for 5%TEI from saturated fatty acids with CHD in the EPIC-CVD case-cohort study: sensitivity analyses

		SFAs to be substituted by:					
		MUFAs		PUFAs		Carbohydrates	
	N cases / total	HR (95% CI)*	I^2 (95% CI)	HR (95% CI)*	I^2 (95% CI)	HR (95% CI)*	I^2 (95% CI)
**Fatal CHD†**	1,614 / 17,688	1.17 (0.89, 1.54)	1 (0-68)	0.98 (0.61, 1.57)	73 (45-87)	1.02 (0.86, 1.21)	18 (0-60)
**Non-fatal CHD†**	8,878 / 24,592	1.14 (0.96, 1.35)	28 (0-68)	1.00 (0.86, 1.16)	41 (0-74)	1.05 (0.96, 1.15)	24 (0-65)
**Excluding the first 2 years of follow-up**	9,479 / 25,637	1.14 (0.96, 1.36)	27 (0-66)	0.98 (0.82, 1.18)	54 (3-78)	1.05 (0.94, 1.16)	36 (0-71)
**Excluding extreme energy intake reporters‡**	10,387 / 26,339	1.14 (0.95, 1.36)	33 (0-69)	0.96 (0.81, 1.15)	54 (3-78)	1.03 (0.93, 1.14)	34 (0-70)
**Winsorized covariates**	10,529 / 26,687	1.11 (0.93, 1.33)	34 (0-70)	0.95 (0.80, 1.13)	54 (2-78)	1.02 (0.92, 1.13)	35 (0-70)
**One-stage analyses§**	10,529 / 26,687	1.03 (0.93, 1.16)	NA	1.02 (0.92, 1.13)	NA	1.01 (0.94, 1.08)	NA

HR, hazard ratio; CHD, coronary heart disease; CI, confidence interval; MUFA, monounsaturated fatty acid; PUFA, polyunsaturated fatty acid; SFA, saturated fatty acid; TEI, total energy intake.

* Hazard ratios (HRs) and 95% confidence intervals (CIs) per 5% higher energy intake from exposure to replace energy from SFAs, analysed within each country separately. Country-specific HRs (95% CIs) were combined in multivariate random-effects meta-analysis to obtain pooled effect estimates and 95% CIs. Models included age as underlying time variable and baseline hazards were stratified by sex. The multivariable-adjusted HR included adjustment age at recruitment (years), centre, energy intake (kcal/day), education (low, medium, high), smoking (never, former, current), physical activity (inactive, moderately inactive, moderately active, active), alcohol intake (0, 0-6, 6-12, 12-13, >24 g/day), dietary fibre (continuous), fruit and vegetable consumption (continuous), body-mass index (continuous), reported history of diabetes, hypertension and hyperlipidemia, and all macronutrients except SFA, i.e. MUFAs, PUFAs, carbohydrates, plant protein, animal protein and mixed-origin protein (all in %TEI).

† France was excluded from analyses of fatal and non-fatal CHD, because there were <10 incident fatal CHD cases, and in analyses of winsorized covariates to aid model convergence. The incidence rates in the subcohort were 23.77 first non-fatal CHD events per 10,000 person-years, and 5.255 fatal CHD events per 10,000 person-years.

‡ Extreme energy intake was defined as total energy intake <500 or >3500 kcal/day for women, and <800 or >4000 kcal/day for men.

§ In one-stage analyses, data from all countries were pooled together in one analysis model.

References:

26. Danesh J, Saracci R, Berglund G, Feskens E, Overvad K, Panico S, Thompson S, Fournier A, Clavel-Chapelon F, Canonico M, Kaaks R, Linseisen J, Boeing H, Pischon T, Weikert C, Olsen A, Tjonneland A, Johnsen SP, Jensen MK, Quiros JR, Svatetz CA, Perez MJ, Larranaga N, Sanchez CN, Iribas CM, Bingham S, Khaw KT, Wareham N, Key T, Roddam A, Trichopoulou A, Benetou V, Trichopoulos D, Masala G, Sieri S, Tumino R, Sacerdote C, Mattiello A, Verschuren WM, Bueno-de-Mesquita HB, Grobbee DE, van der Schouw YT, Melander O, Hallmans G, Wennberg P, Lund E, Kumle M, Skeie G, Ferrari P, Slimani N, Norat T, Riboli E and Heart E. EPIC-Heart: the cardiovascular component of a prospective study of nutritional, lifestyle and biological factors in 520,000 middle-aged participants from 10 European countries. *European journal of epidemiology*. 2007;22:129-41.

29. InterAct C, Langenberg C, Sharp S, Forouhi NG, Franks PW, Schulze MB, Kerrison N, Ekelund U, Barroso I, Panico S, Tormo MJ, Spranger J, Griffin S, van der Schouw YT, Amiano P, Ardanaz E, Arriola L, Balkau B, Barricarte A, Beulens JW, Boeing H, Bueno-de-Mesquita HB, Buijsse B, Chirlaque Lopez MD, Clavel-Chapelon F, Crowe FL, de Lauzon-Guillan B, Deloukas P, Dorronsoro M, Drogan D, Froguel P, Gonzalez C, Grioni S, Groop L, Groves C, Hainaut P, Halkjaer J, Hallmans G, Hansen T, Huerta Castano JM, Kaaks R, Key TJ, Khaw KT, Koulman A, Mattiello A, Navarro C, Nilsson P, Norat T, Overvad K, Palla L, Palli D, Pedersen O, Peeters PH, Quiros JR, Ramachandran A, Rodriguez-Suarez L, Rolandsson O, Romaguera D, Romieu I, Sacerdote C, Sanchez MJ, Sandbaek A, Slimani N, Sluijs I, Spijkerman AM, Teucher B, Tjonneland A, Tumino R, van der AD, Verschuren WM, Tuomilehto J, Feskens E, McCarthy M, Riboli E and Wareham NJ. Design and cohort description of the InterAct Project: an examination of the interaction of genetic and lifestyle factors on the incidence of type 2 diabetes in the EPIC Study. *Diabetologia*. 2011;54:2272-82.

Figure S1. Number of participants included in analyses of the association of dietary fatty acids and incident coronary heart disease: EPIC-CVD case-cohort study.

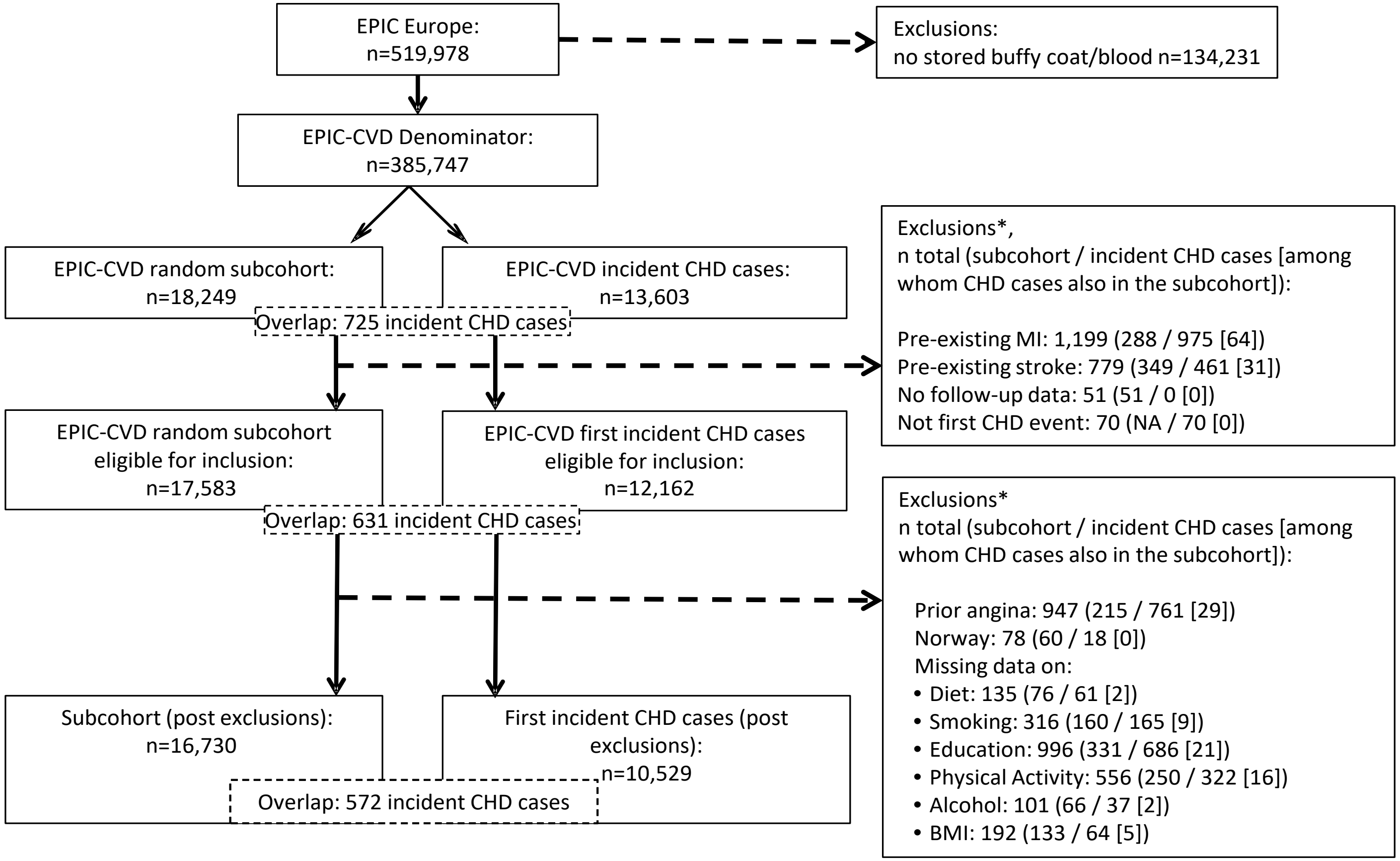

BMI, body mass index; CHD, coronary heart disease; CVD, cardiovascular disease; EPIC, European Prospective Investigation into Cancer and Nutrition; MI, myocardial infarction;*The number of exclusions due to different causes within each box may overlap.
Norway was excluded due to small sample size.

Figure S2. Distribution of dietary fatty acid intake levels across countries in the EPIC-CVD case-cohort study subcohort (n=16,730).

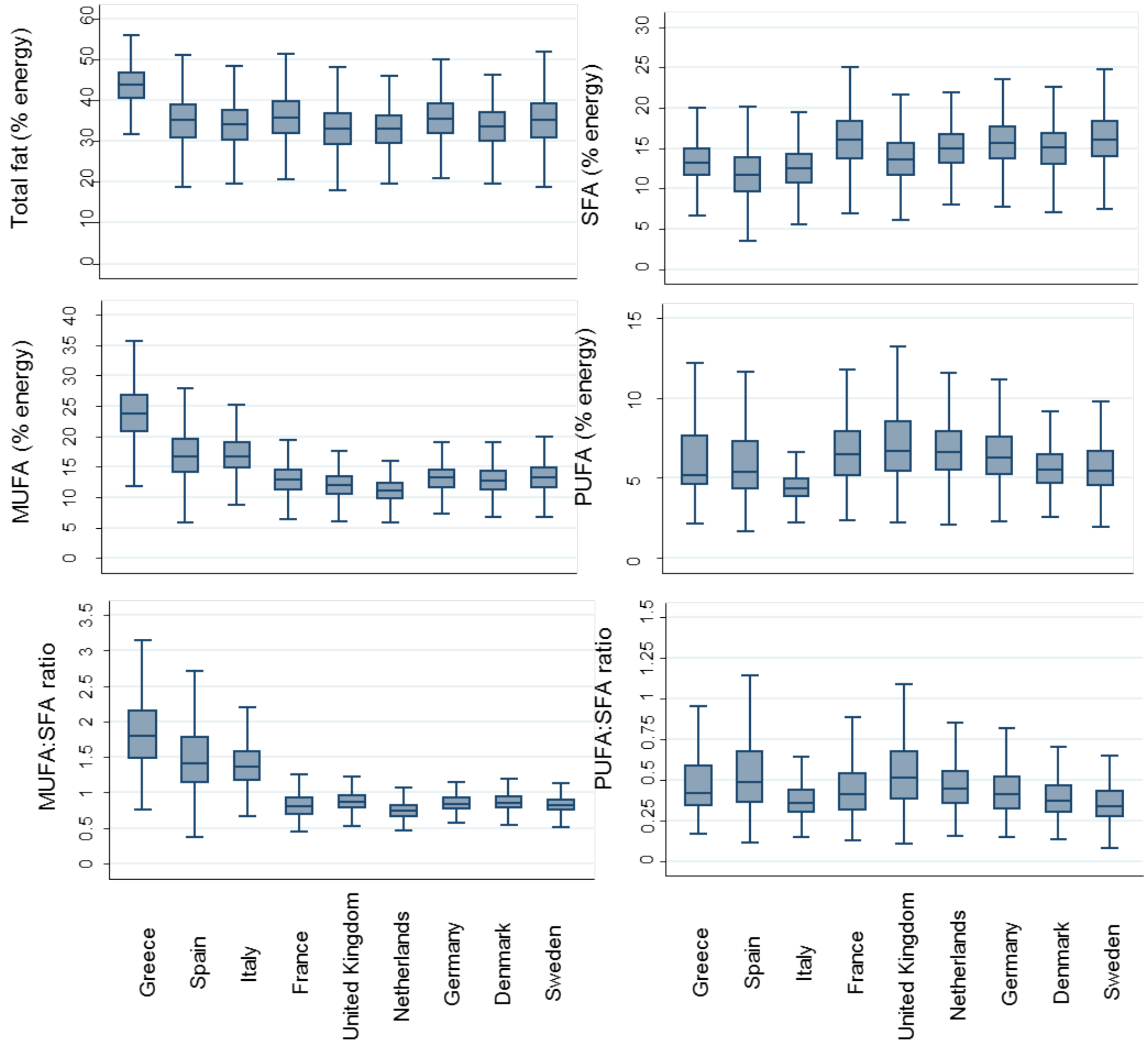

MUFAs, monounsaturated fatty acids; PUFAs, polyunsaturated fatty acids; SFAs, saturated fatty acids; Boxes present 25th percentile, median, 75th percentile, whiskers are the 25th percentile minus 1·5* the interquartile range (lower) and the 75th percentile plus 1·5*the interquartile range (upper). Fatty acids were expressed in percentage of total energy intake, and their distributions evaluated within the subcohort in each country.

Figure S3. Pearson partial correlation coefficients of dietary fatty acids with food groups in the EPIC-CVD case-cohort study subcohort (n=16,730).

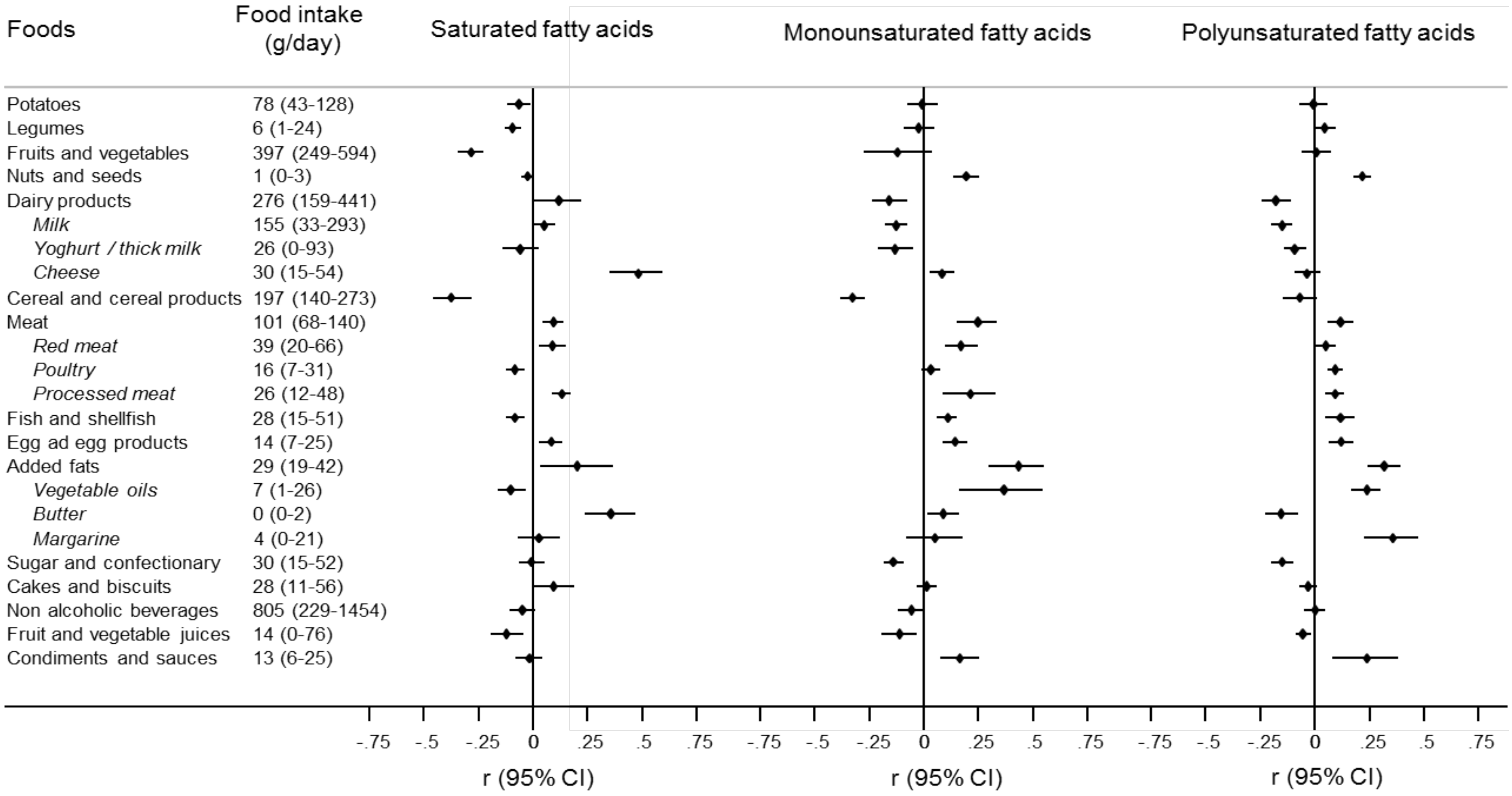

Food intake levels are presented in median (interquartile range). Country-specific correlations r (95% confidence interval [CI]) were adjusted for age, sex, energy intake and body mass index. Fisher's Z transformation was then used to transform country-specific correlation coefficients r (95% CIs) to obtain a normally distributed variable, i.e. using z = 0.5*ln((1+r)/1-r)). Z-transformed correlation coefficients r (95% CIs) were subsequently pooled using random effects meta-analysis, and back-transformation was applied to obtain an overall correlation coefficient r (95% CI). Dietary saturated, monounsaturated and polyunsaturated fatty acids were expressed in % total energy intake. Polyunsaturated fatty acids were log-transformed to normalize the distribution. Food group `vegetable oils` includes all vegetable oils evaluated by country -specific dietary questionnaires, including olive oil where applicable.

Figure S4. Associations with CHD of dietary saturated, monounsaturated and polyunsaturated fatty acids in the EPIC-CVD case-cohort study, analysed with the energy residual method.

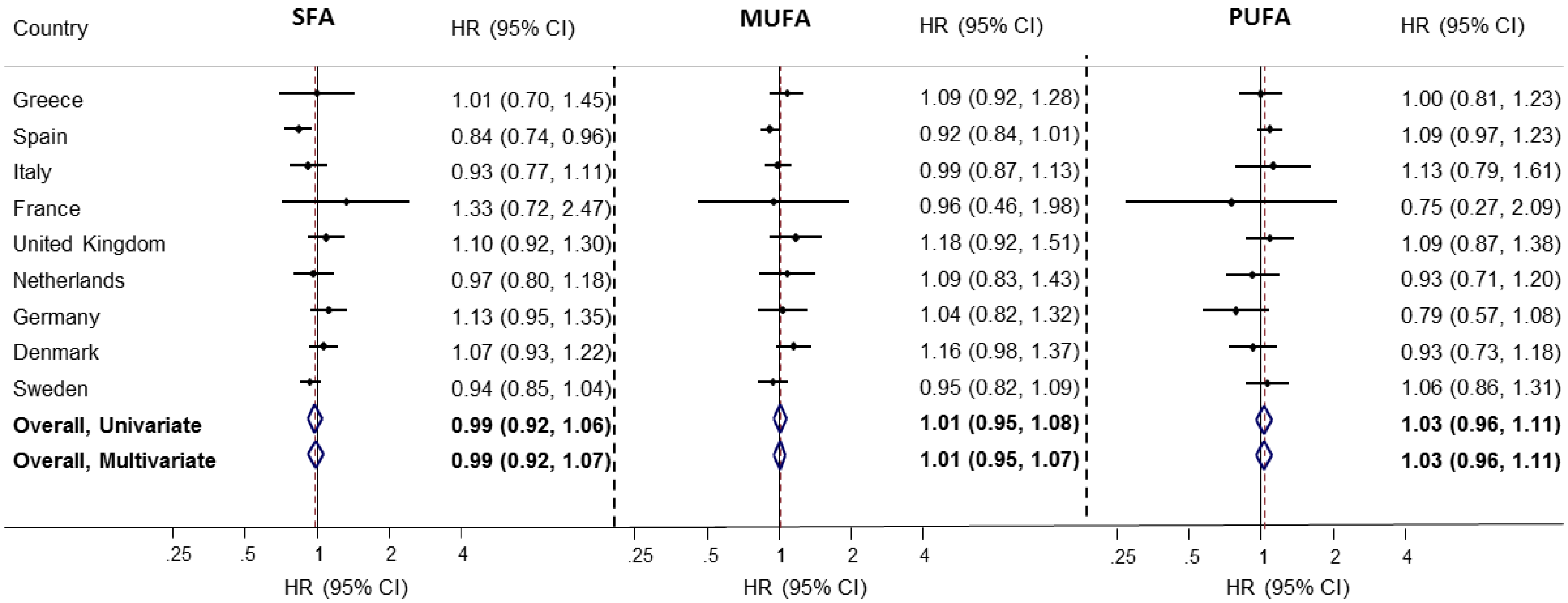

Hazard ratios (HRs) and 95% confidence intervals (CIs) for each 10 energy-adjusted g/day higher intake from saturated fatty acids (SFAs), monounsaturated fatty acids (MUFAs) and polyunsaturated fatty acids (PUFAs) were analysed within each country separately, with age as the underlying time variable and the baseline hazard stratified by sex. Country-specific HRs (95% CIs) were combined in univariate and multivariate random-effects meta-analysis to obtain pooled effect estimates and 95% CIs. The multivariable-adjusted HR included adjustment for age at recruitment (years), centre, energy intake (kcal/day), education (low, medium, high), smoking (never, former, current), physical activity (inactive, moderately inactive, moderately active, active), alcohol intake (0, 0-6, 6-12, 12-13, >24 g/day), dietary fibre (g/day, continuous), fruit and vegetable consumption (g/day, continuous), body-mass index (kg/m^2, continuous), reported history of diabetes, hypertension and hyperlipidaemia.

Figure S5. Associations with CHD of dietary saturated, monounsaturated and polyunsaturated fatty acids in the EPIC-CVD case-cohort study, analysed with the energy partition model.

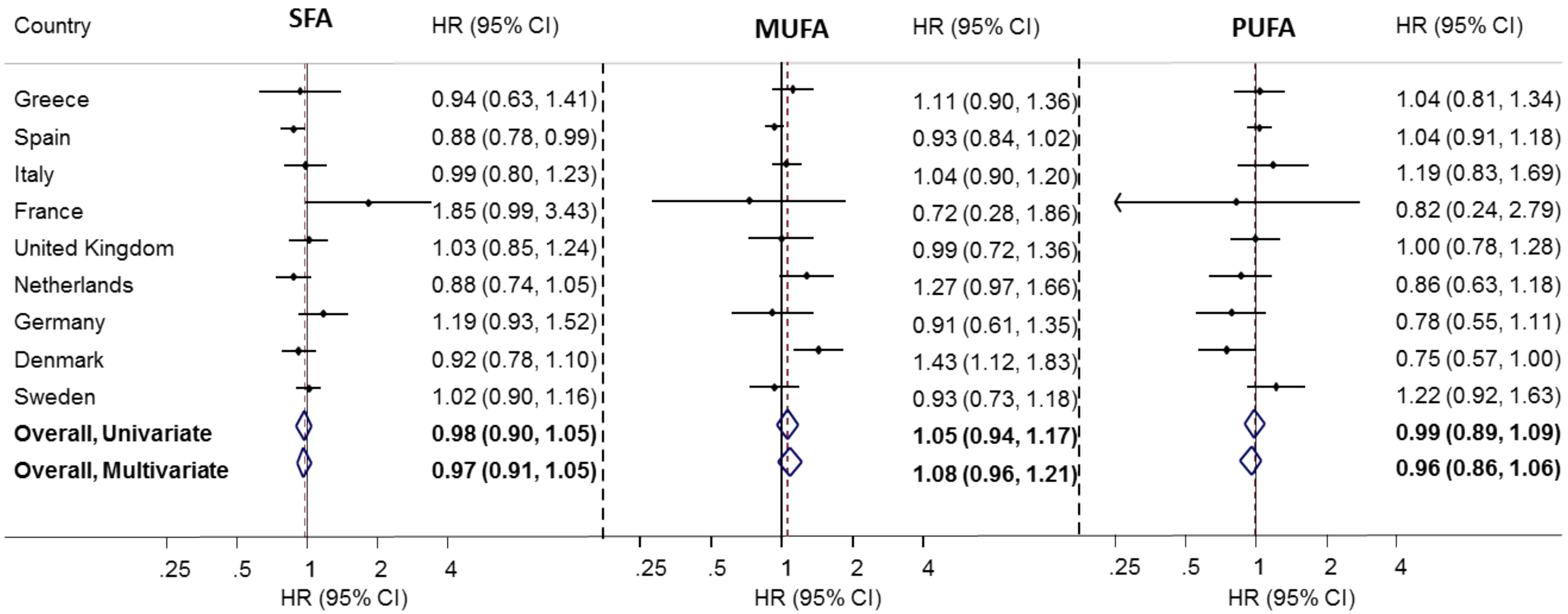

Hazard ratios (HRs) and 95% confidence intervals (CIs) for each 10 g/day higher intake from saturated fatty acids (SFAs), monounsaturated fatty acids (MUFAs) and polyunsaturated fatty acids (PUFAs) were analysed within each country separately, with age as the underlying time variable and the baseline hazard stratified by sex. Country-specific HRs (95% CIs) were combined in univariate and multivariate random-effects meta-analysis to obtain pooled effect estimates and 95% CIs. The multivariable-adjusted HRs included adjustment for age at recruitment (years), centre, education (low, medium, high), smoking (never, former, current), physical activity (inactive, moderately inactive, moderately active, active), alcohol intake (0, 0-6, 6-12, 12-13, >24 g/day), dietary fibre (g/day, continuous), fruit and vegetable consumption (g/day, continuous), body-mass index (kg/m^2, continuous), reported history of diabetes, hypertension and hyperlipidaemia, and SFAs, MUFAs, PUFAs, carbohydrates, . animal-derived protein, plant-derived protein, and mixed-origin protein (all per 10 g/day, included simultaneously in the model).
